# Long-term Effect of Face-to-Face vs Virtual Reality Cardiopulmonary Resuscitation (CPR) Training on Willingness to Perform CPR, Retention of Knowledge, and Dissemination of CPR Awareness: A Secondary Analysis of a Randomized Clinical Trial

**DOI:** 10.1001/jamanetworkopen.2022.12964

**Published:** 2022-05-19

**Authors:** Joris Nas, Jos Thannhauser, Lara S. F. Konijnenberg, Robert-Jan M. van Geuns, Niels van Royen, Judith L. Bonnes, Marc A. Brouwer

**Affiliations:** 1 Department of Cardiology, Radboud University Medical Center, Nijmegen, the Netherlands

## Abstract

**Importance:**

Increased bystander cardiopulmonary resuscitation (CPR) is essential to improve survival after cardiac arrest. Although most studies focus on technical CPR skills, the randomized Lowlands Saves Lives trial prespecified a follow-up survey on other important aspects that affect the widespread performance of CPR.

**Objective:**

To investigate bystander willingness to perform CPR on a stranger, theoretical knowledge retention, and dissemination of CPR awareness 6 months after undergoing short face-to-face and virtual reality (VR) CPR trainings.

**Design, Setting, and Participants:**

A prespecified 6-month posttraining survey was conducted among 320 participants in the Lowlands Saves Lives trial, a randomized comparison between 20-minute face-to-face, instructor-led CPR training and VR training. Participants were recruited at the Lowlands music festival, with a designated area to conduct scientific projects (August 16-18, 2019; the Netherlands). Statistical analysis was performed from March 1, 2020, to July 31, 2021.

**Interventions:**

Two standardized 20-minute protocols on CPR and automated external defibrillator use: instructor-led face-to-face training using CPR manikins or VR training using the Resuscitation Council (UK)–endorsed Lifesaver VR smartphone application and a pillow to practice compressions.

**Main Outcomes and Measures:**

Primary outcomes were willingness to perform CPR on a stranger, theoretical knowledge retention, and dissemination of CPR awareness as reported by the entire cohort. As secondary analyses, the results of the 2 training modalities were compared.

**Results:**

Of 381 participants, 320 consented to this follow-up survey; 188 participants (115 women [61%]; median age, 26 years [IQR, 22-32 years]) completed the entire survey and were accordingly included in the secondary analysis. The overall proportion of participants willing to perform CPR on a stranger was 77% (144 of 188): 81% (79 of 97) among face-to-face participants and 71% (65 of 91) among VR participants (*P* = .02); 103 participants (55%) reported feeling scared to perform CPR (*P* = .91). Regarding theoretical knowledge retention, a median of 7 (IQR, 6-8) of 9 questions were answered correctly in both groups (*P* = .81). Regarding dissemination of CPR awareness, 65% of participants (123 of 188) told at least 1 to 10 family members or friends about the importance of CPR, and 15% (29 of 188) had participated in certified, instructor-led training at the time of the survey, without differences between groups.

**Conclusions and Relevance:**

In this 6-month posttraining survey, young adult participants of short CPR training modules reported high willingness (77%) to perform CPR on a stranger, with slightly higher rates for face-to-face than for VR participants. Theoretical knowledge retention was good, and the high dissemination of awareness suggests that these novel CPR training modules staged at a public event are promising sensitizers for involvement in CPR, although further challenges include mitigating the fear of performing CPR.

**Trial Registration:**

ClinicalTrials.gov Identifier: NCT04013633

## Key Points

**Question:**

What is the effect of 20-minute cardiopulmonary resuscitation (CPR) training modules, staged during a public event, on willingness to perform CPR, retention of knowledge, and dissemination of CPR awareness?

**Findings:**

This 6-month posttraining survey of 188 participants in a randomized clinical trial on face-to-face vs virtual reality CPR training found high willingness to perform CPR on a stranger (77%), with slightly higher rates for face-to-face training than virtual reality (81% vs 71%). Regardless of training method, the median theoretical knowledge score was 7 out of 9 questions answered correctly, 15% of participants completed a certified CPR course after the study, and each participant informed at least 1 to 10 friends or relatives about the importance of CPR.

**Meaning:**

In addition to imparting technical CPR skills, short CPR training modules may lead to high willingness to perform CPR, good theoretical knowledge of CPR, and high dissemination of CPR awareness.

## Teaser

This secondary analysis of a randomized clinical trial investigates bystander willingness to perform cardiopulmonary resuscitation on a stranger, theoretical knowledge retention, and dissemination of cardiopulmonary resuscitation awareness 6 months after undergoing short face-to-face or virtual reality training.

## Introduction

Annually, more than 300 000 patients experience an out-of-hospital cardiac arrest in the United States alone, with a mean mortality rate of 75% to 90%.^1^ In Europe, the incidence of out-of-hospital cardiac arrest is 56 individuals per 100 000 population per year, with a mean rate of survival to hospital discharge of 8%.^2^ Early bystander cardiopulmonary resuscitation (CPR) is a key determinant of survival but is not performed in approximately 40% of individuals experiencing out-of-hospital cardiac arrest.^3,4^ To improve cardiac arrest outcomes, training of additional volunteers is essential.^5^ As such, education has been identified as one of the top priorities for cardiac arrest research, with the new guidelines reemphasizing its importance.^6,7,8^

In recent years, many studies have been conducted to investigate new and innovative training methods. Most of these studies focus on measuring technical CPR skills after training.^9^ Although technical CPR skills are indispensable, previous studies have also identified other factors that are important for the widespread performance of CPR,^10,11,12^ such as fear of doing harm or uncertainty about CPR performance, but data on these factors in relation to recent training are scarce. Another important challenge is dissemination of awareness of the importance of CPR, which may help to increase bystander willingness to perform CPR. Additional information on the effect of recent training on these aforementioned factors may provide insights that can be used to improve training and subsequently increase bystander willingness to perform CPR.

A recent randomized clinical trial of short CPR modules was conducted among 381 participants during a 3-day music festival, a unique setting to sensitize young people to CPR and create awareness for the importance of CPR and CPR training.^13^ The present study reports on the prespecified 6-month follow-up survey of that trial and focuses on bystander willingness to perform CPR, knowledge retention, and dissemination of CPR awareness.

## Methods

The Lowlands Saves Lives trial is registered at ClinicalTrials.gov (NCT04013633). This secondary analysis followed the Consolidated Standards of Reporting Trials (CONSORT) reporting guideline for randomized clinical trials. All participants provided written informed consent. The protocol was approved by the Research Ethics Committee of the Radboud University Medical Center. The trial protocol is available in Supplement 1. This posttraining survey was a prespecified substudy of the Lowlands Saves Lives trial, a 1:1 randomized clinical trial comparing CPR quality between face-to-face and Lifesaver virtual reality (VR) CPR training as previously described.^13,14^

### Objective and Outcome Measures

The primary aim of this study was to assess the effect of 20-minute CPR training on willingness to perform CPR, knowledge retention, and dissemination of CPR awareness for all survey respondents. Secondarily, we compared the results between the 2 training modules (ie, face-to-face training compared with VR training).

Primary outcomes were the answers to the questions regarding willingness to perform CPR, theoretical knowledge of CPR, and dissemination of CPR skills. Secondary outcomes were the answers to all other questions. The entire survey can be found in eTable 1 in Supplement 2.

### Study Population

Participants were recruited at Lowlands Science, a section of the Lowlands music festival (August 16-18, 2019, Biddinghuizen, the Netherlands) dedicated exclusively to conducting scientific research (for an overview of the study area, see eFigure 1 in Supplement 2). The scope of the Lowlands music festival is broader than that of most music festivals because it aims to connect the world of music with that of culture, theatre, and innovative science. During the Lowlands music festival, the science section was a designated area on the festival grounds with its own entrance, with a specific focus on scientific research. Here, festival participants could enter freely (no additional charge) to browse and get updates on and/or participate in new scientific projects. The science section was near the music stages but physically separated from the rest of the festival. Participants were eligible if they were adults (≥18 years) and deemed physically and mentally capable of participating in CPR training and taking the CPR test. All participants completed a baseline survey regarding demographic characteristics and previous CPR training and experience. Because alcohol consumption is common during music festivals, we measured the alcohol level in each participant using a law-enforcement grade alcohol breathalyzer test (AlcoTrue P, Bluepoint Medical). We used the European unit of measurement (ie, per mille [‰]), which differs from the unit used in the United States (ie, percentage [%]) (Table 1).

**Table 1.** Baseline Characteristics of Participants Who Received Face-to-Face vs Lifesaver VR Training

Characteristic	No.	Participants, No. (%)		*P* value^a^
		Face-to-face group (n = 97)	Lifesaver VR group (n = 91)	
Female sex	188	62 (64)	53 (58)	.43
Age, median (IQR), y	188	25 (22-31)	27 (23-32)	.21
Weight, median (IQR), kg	186	71 (62-80)	71 (65-82)	.50
University education	188	44 (45)	44 (48)	.68
Health care professional	187	33 (34)	17 (19)	.02
Alcohol level, median (IQR), ‰^b^	188	0.0 (0.0-0.25)	0.0 (0.0-0.34)	.47
Alcohol level ≥0.5‰^b^	188	8 (8)	16 (18)	.06
Use of drugs or narcotics ≤24 h	184	25 (27)	18 (20)	.29
Previous CPR course ≤2 y	175	18 (21)	16 (18)	.73
Ever witnessed a cardiac arrest	188	14 (14)	14 (15)	.86

Abbreviations: CPR, cardiopulmonary resuscitation; VR, virtual reality.

^a^ For comparison between groups.

^b^ Alcohol levels are reported using the European unit of measure (‰), which is a factor of 10 higher than the US unit of measurement (%).

### Intervention

We used the Castor Electronic Data Capture system to perform randomization, using a random block size randomization algorithm. After randomization, participants completed one of the following two 20-minute training methods: face-to-face training by experienced European Resuscitation Council–certified instructors or VR training using the UK Resuscitation Council–endorsed Lifesaver smartphone application.

In the face-to-face group, there was 1 certified CPR instructor for every 5 participants; instructors provided education on technical CPR skills. Chest compressions were practiced on Laerdal Little Anne CPR manikins, and automated external defibrillator skills were practiced using Zoll Trainer 3 automated external defibrillators. In the VR group, all participants completed the same VR CPR scenario, and chest compressions were practiced on a pillow. The standardized training protocols were based on the European Resuscitation Council guidelines and have been published previously.^13,14^ Both training modules were aimed at teaching technical CPR skills.

Before and after the technical training sessions, the importance of CPR was emphasized by the physicians of our research team, and there were several posters and billboards about CPR in the study area. In addition, study personnel were available to answer any additional questions regarding CPR.

### Follow-up Assessment

As specified in our study protocol, the subset of participants who provided written informed consent for follow-up assessment were asked to take a digital survey 6 months after the CPR training, with several reminders sent after the initial request to participate.^14^ The survey was conducted using Castor Electronic Data Capture.

The development process of the survey was based on previously reported methods.^15^ The survey tool was created by the main investigators (J.N., J.T., and M.A.B.) and finalized after an iterative process. The survey comprised multiple choice questions, including “attention questions” to check that participants were not simply clicking through the survey without reading the questions. Consistent with other surveys, this strategy is used to guarantee quality data and serves as an internal check to ensure full respondent attention and participation.^15^ If the respondent failed these attention questions, his or her survey was excluded from further analysis.

### Statistical Analysis

Statistical analysis was performed from March 1, 2020, to July 31, 2021. The parameters of the study were assessed for normal distribution and accordingly reported as mean (SD) values or median values with IQRs. Continuous data were compared between the 2 study groups (face-to-face training vs VR training) using the *t* test or the Mann-Whitney test, and categorical variables were reported as numbers and percentages and compared using the χ^2^ test or the Fisher exact test, whichever was appropriate. All *P* values were from 2-sided tests and results were deemed statistically significant at *P* < .05. Analyses were performed using SPSS, version 25 (IBM Corp).

## Results

### Participant Flow and Recruitment

In total, 381 participants were included in the Lowlands Saves Lives trial, of whom 320 provided written informed consent for follow-up and 195 responded to our request. Of these 320 participants, 188 completed the entire survey and were accordingly included in the present analysis (for a complete overview, see CONSORT flowchart in eFigure 2 in Supplement 2).

There were no statistically significant differences in baseline characteristics or in CPR quality between the included and excluded participants, except for the proportion of health care professionals (27% [50 of 188] among included participants and 15% [29 of 193] among excluded participants; *P* = .005) (eTable 2 in Supplement 2). There was a nonsignificantly lower proportion of participants with an alcohol level of 0.5‰ or more among included vs excluded participants (13% [24 of 188] vs 20% [39 of 193]; *P* = .05).

### Baseline Characteristics

Of the 188 respondents, 97 had participated in the face-to-face training and 91 had participated in the VR training. In total, 115 participants (61%) were women, and the median age of the participants was 26 years (IQR, 22-32 years). Baseline characteristics were similar between the 2 study groups, except for the proportion of health care professionals, which was 34% (33 of 97) in the face-to-face group and 19% (17 of 91) in the VR group (*P* = .02) (Table 1). The median alcohol level was 0.0‰ (IQR, 0.0‰-0.25‰) in the face-to-face group and 0.0‰ (IQR, 0.0‰-0.34‰) in the VR group (*P* = .47). There was a nonsignificantly lower proportion of participants with an alcohol level of 0.5‰ or more in the face-to-face group vs the VR group (8% [8 of 97] vs 18% [16 of 91]; *P* = .06).

### Primary Outcome Measures

The overall proportion of participants who would start CPR on a stranger was 77% (144 of 188) (81% [79 of 97] in the face-to-face group vs 71% [65 of 91] in the VR group; *P* = .02), and 87% (163 of 188) indicated that they would start CPR on a relative or friend (91% [88 of 97] in the face-to-face group vs 82% [75 of 91] in the VR group; *P* = .05) (Table 2). A total of 103 participants (55%) reported feeling scared to perform CPR (*P* = .91). The overall median number of the theoretical knowledge questions that were answered correctly was 7 (IQR, 6-8) of 9 questions (7 [IQR, 6-8] in the face-to-face group and 7 [IQR, 6-8] in the VR group; *P* = .81) (Table 3). In total, 95% of participants (178 of 188) told their family or acquaintances about study participation (92% [89 of 97] in the face-to-face group and 98% [89 of 91] in the VR group; *P* = .07), 65% (123 of 188) told them about the importance of CPR in general (64% [62 of 97] in the face-to-face group vs 67% [61 of 91] in the VR group; *P* = .87), and 59% (110 of 188) told them about the importance of CPR training (62% [60 of 97] in the face-to-face group vs 55% [50 of 91] in the VR group; *P* = .45), with 50% of participants (94 of 188) indicating that they each informed 1 to 10 friends or acquaintances about the importance of CPR in general (46% [45 of 97] in the face-to-face group vs 54% [49 of 91] in the VR group; *P* = .54) (Table 4).^16^

**Table 2.** Self-reported Attitude and Willingness

Question and answers	Participants, No. (%)			*P* value^a^
	All (N = 188)	Face-to-face group (n = 97)	Lifesaver VR group (n = 91)	
Do you feel capable to perform CPR after study participation?				
Yes, because of study participation	46 (25)	26 (27)	20 (22)	.87
Yes, I already did before the study	72 (38)	37 (38)	35 (39)	
Yes, because I did receive CPR training after the study	8 (4)	4 (4)	4 (4)	
No	62 (33)	30 (31)	32 (35)	
If you were to witness a cardiac arrest of an unknown person, would you start CPR?				
Yes	144 (77)	79 (81)	65 (71)	.02
No	7 (4)	0	7 (8)	
Do not know	37 (20)	18 (19)	19 (21)	
If you were to witness a cardiac arrest of a family member or friend, would you start CPR?				
Yes	163 (87)	88 (91)	75 (82)	.05
No	5 (3)	0	5 (6)	
Do not know	20 (11)	9 (9)	11 (12)	
I have the feeling that I am capable of helping somebody experiencing cardiac arrest				
Strongly disagree	5 (3)	2 (2)	3 (3)	.06
Disagree	22 (12)	8 (8)	14 (15)	
Neutral	39 (21)	17 (18)	22 (24)	
Agree	92 (49)	48 (50)	44 (48)	
Strongly agree	30 (16)	22 (23)	8 (9)	
I would be scared to perform CPR				
Strongly disagree	11 (6)	7 (7)	4 (4)	.91
Disagree	28 (15)	13 (13)	15 (17)	
Neutral	46 (25)	23 (24)	23 (25)	
Agree	86 (46)	45 (46)	41 (45)	
Strongly agree	17 (9)	9 (9)	8 (9)	
I do not want to perform mouth-to-mouth ventilations on a stranger				
Strongly disagree	37 (20)	19 (20)	18 (20)	.88
Disagree	73 (39)	37 (38)	36 (40)	
Neutral	53 (28)	30 (31)	23 (25)	
Agree	20 (11)	9 (9)	11 (12)	
Strongly agree	5 (3)	2 (2)	3 (3)	

Abbreviations: CPR, cardiopulmonary resuscitation; VR, virtual reality.

^a^ For comparisons between groups.

**Table 3.** Theoretical Capability to Perform CPR

Questions	Answers	Participants, No. (%)			*P* value^a^
		All (N = 188)	Face-to-face group (n = 97)	Lifesaver VR group (n = 91)	
**Q1: You are on the platform and see a person lying on the floor. What do you do first?**					
Correct	Check for safety	147 (78)	75 (77)	72 (79)	.96
Incorrect	Check for breathing	15 (8)	8 (8)	7 (8)	
	Check for response	26 (14)	14 (14)	12 (13)	
**Q2: To check if the person is conscious, you have to do the following:**					
Correct	Gently shake his shoulders and ask loudly, “Sir can you hear me?”	159 (85)	84 (87)	75 (82)	.71
Incorrect	Gently shake his head and ask loudly, “Sir can you hear me?”	4 (2)	2 (2)	2 (2)	
	Gently shake his shoulders, pinch his arm, and scream, “Sir can you hear me?”	25 (13)	11 (11)	14 (15)	
**Q3: The person on the platform does not respond. What do you do immediately?**					
Correct	Call emergency medical services (112 in the Netherlands)	153 (81)	79 (81)	74 (81)	>.99
Incorrect	Open the airway	33 (18)	17 (18)	16 (18)	
	Start chest compressions	2 (1)	1 (1)	1 (1)	
**Q4: How would you make sure that the airway of the person is opened?**					
Correct	Correct chin lift	151 (80)	81 (84)	70 (77)	.46
Incorrect	Pinch the nose with 1 hand and place the other hand under the chin to tilt the head	27 (14)	11 (11)	16 (18)	
	Put 1 hand on the forehead to tilt the head backward	10 (5)	5 (5)	5 (6)	
**Q5: If the person is not breathing normally, what do you do first?**					
Correct	Start chest compressions	147 (78)	77 (79)	70 (77)	.91
Incorrect	Put him on his side	22 (12)	11 (11)	11 (12)	
	Call emergency medical services and wait for the ambulance to arrive	19 (10)	9 (9)	10 (11)	
**Q6: How deep should you perform chest compressions when performing CPR?**					
Correct	5-6 cm	122 (65)	64 (66)	58 (64)	.72
Incorrect	≥7 cm	29 (15)	13 (13)	16 (18)	
	4-5 cm	37 (20)	20 (21)	17 (19)	
**Q7: How fast should you perform chest compressions?**					
Correct	2 compressions per second	108 (57)	52 (54)	56 (62)	.54
Incorrect	1 compression per second	78 (42)	44 (45)	34 (37)	
	1 compression per 10 s	2 (1)	1 (1)	1 (1)	
**Q8: When should you stop chest compressions to give mouth-to-mouth ventilations?**					
Correct	After 30 compressions	168 (89)	88 (91)	80 (88)	.55
Incorrect	After 10 compressions	16 (9)	8 (8)	8 (9)	
	After about 1 min of compressions	4 (2)	1 (1)	3 (3)	
**Q9: How do you place the AED pads?**					
Correct	Figure 1	126 (67)	62 (64)	64 (70)	.52
Incorrect	Figure 2	18 (10)	9 (9)	9 (10)	
	Figure 3	44 (23)	26 (27)	18 (20)	
**Overall**					
Total score (each correctly answered question is 1 point)^b^	NA	7 (6-8)	7 (6-8)	7 (6-8)	.81

Abbreviations: AED, automated external defibrillator; CPR, cardiopulmonary resuscitation; NA, not applicable; VR, virtual reality.

^a^ For comparisons between groups.

^b^ Total scores are median values (IQRs).

**Table 4.** Action Taken After Study Participation

Question and answers	Participants, No. (%)			
	All (N = 188)	Face-to-face group (n = 97)	Lifesaver VR group (n = 91)	*P* value^a^
**Part I: self**				
After participating in the study, did you look for more information on CPR (eg, online)?				
Do not know	6 (3)	5 (5)	1 (1)	.24
No	132 (70)	65 (67)	67 (74)	
Yes	50 (27)	27 (28)	23 (25)	
On CPR in general	34 (18)	19 (20)	15 (17)	.58
On Lifesaver VR	6 (3)	1 (1)	5 (6)	.11
How to apply for a CPR course	23 (12)	10 (10)	13 (14)	.41
How to become a registered volunteer	6 (3)	4 (4)	2 (2)	.68
Where the nearest AED is	14 (7)	8 (8)	6 (7)	.67
Other	5 (3)	5 (5)	0	.06
Did you follow an instructor-led CPR training after study participation?				
Yes	29 (15)	16 (17)	13 (14)	.68
No	159 (85)	81 (84)	78 (86)	
I feel capable enough	26 (14)	10 (10)	16 (18)	.55
It takes too much time	10 (5)	5 (5)	5 (5)	
It is too expensive	27 (14)	15 (15)	12 (13)	
Not yet, but planning to	50 (27)	29 (30)	21 (23)	
Other	46 (25)	22 (23)	24 (26)	
Did you download the Lifesaver application after study participation?				
Yes, downloaded and planning to train	1 (0.5)	0	1 (1)	.96
Yes, also trained	1 (0.5)	1 (1)	0	
No	186 (99)	96 (99)	90 (99)	
Planning to	32 (17)	16 (17)	16 (18)	.85
I feel capable enough	34 (18)	20 (21)	14 (15)	.35
Do not know where to find it	28 (15)	13 (13)	15 (17)	.55
It takes too much time	3 (2)	1 (1)	2 (2)	.61
VR goggles too expensive	34 (18)	17 (18)	17 (19)	.84
Do not know how to get VR goggles	20 (11)	8 (8)	12 (13)	.27
It is not pleasant to use	3 (2)	0	3 (3)	.11
Other	67 (36)	38 (39)	29 (32)	.30
Did you register on a website for CPR volunteers?^b^				
Yes, after participation in the study	4 (2)	4 (4)	0	.28
Yes, but I already was registered before study participation	24 (13)	13 (13)	11 (12)	
No, but I am planning to	31 (17)	15 (16)	16 (18)	
No, I do not want to	52 (28)	29 (30)	23 (25)	
No, I do not know what that is	77 (41)	36 (37)	41 (45)	
Did you witness a cardiac arrest after study participation?				
Yes, professionally	15 (8)	7 (7)	8 (9)	.75
Yes, nonprofessionally	3 (2)	1 (1)	2 (2)	
No	170 (90)	89 (92)	81 (89)	
Did you perform CPR after study participation?				
Yes, professionally	12 (6)	5 (5)	7 (8)	.45
Yes, nonprofessionally	1 (0.5)	0	1 (1)	
No	175 (93)	92 (95)	83 (91)	
**Part II: family or acquaintances**				
Did you tell family or acquaintances about participating in the study?				
Do not know	0	0	0	.07
No	10 (5)	8 (8)	2 (2)	
Yes	178 (95)	89 (92)	89 (98)	
1-10 Persons	130 (69)	67 (69)	63 (69)	.77
11-20 Persons	40 (21)	18 (19)	22 (24)	
>20 Persons	8 (4)	4 (4)	4 (4)	
Did you tell family or acquaintances about the importance of CPR in general?				
Do not know	14 (7)	8 (8)	6 (7)	.87
No	51 (27)	27 (28)	24 (26)	
Yes	123 (65)	62 (64)	61 (67)	
1-10 Persons	94 (50)	45 (46)	49 (54)	.54
11-20 Persons	23 (12)	14 (14)	9 (10)	
>20 Persons	6 (3)	3 (3)	3 (3)	
Did you tell family or acquaintances about the importance of CPR training?				
Do not know	18 (10)	7 (7)	11 (12)	.45
No	60 (32)	30 (31)	30 (33)	
Yes	110 (59)	60 (62)	50 (55)	
1-10 Persons	90 (48)	48 (49)	42 (46)	.85
11-20 Persons	12 (6)	7 (7)	5 (5)	
>20 Persons	8 (4)	5 (5)	3 (3)	
Did your family or acquaintances take action in response to your participation?				
Do not know	77 (41)	36 (37)	41 (45)	.51
No	101 (54)	56 (58)	45 (50)	
Yes	10 (5)	5 (5)	5 (6)	
Looked for information on CPR course	6 (3)	3 (3)	3 (3)	.94
Applied for CPR course	4 (2)	2 (2)	2 (2)	.95
Took a CPR course	4 (2)	0	4 (4)	.04

Abbreviations: AED, automated external defibrillator; CPR, cardiopulmonary resuscitation; VR, virtual reality.

^a^ For comparisons between groups.

^b^ This refers to a Dutch website^16^ that registers civilian volunteers, who can then be summoned to the site of a cardiac arrest through a text message–based alert system, activated by the emergency medical services dispatch center.

### Secondary Outcome Measures

#### General Impression of the Study and Intervention

The results of these questions can be found in the Figure. Overall, 84% of the participants (157 of 188) marked the general experience with the study as positive (87% [84 of 97] in the face-to-face group and 80% [73 of 91] in the VR group; *P* = .12). The training was rated as positive by 69% of all participants (130 of 188), with a higher proportion among the face-to-face group vs the VR group (81% [79 of 97] vs 56% [51 of 91]; *P* < .001). Seventy-six percent (142 of 188) of all participants felt positive about receiving CPR training at the festival (74% [72 of 97] in the face-to-face group and 77% [70 of 91] in the VR group; *P* = .31).

**Figure.**
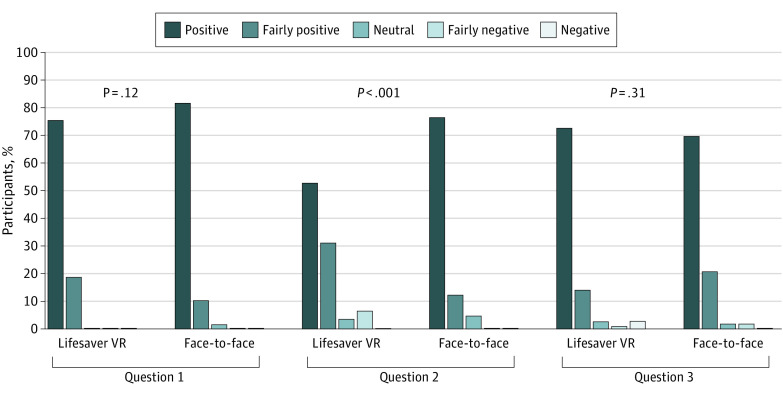
General Impression of the Study and Intervention General impression of the study and the intervention among those trained using Lifesaver virtual reality (VR) compared with those who underwent face-to-face training. Question 1: How would you rate your overall experience with the study? Question 2: How would you rate the training that you followed? Question 3: How do you feel about receiving cardiopulmonary resuscitation training at a festival?

### Self-reported Willingness to Perform CPR

The answers and questions regarding self-reported willingness to perform CPR are shown in Table 2. It can be seen that 65% of all participants (122 of 188) agreed or strongly agreed with the statement that they would feel capable to perform CPR, with a nonsignificantly higher proportion in the face-to-face group than in the VR group (72% [70 of 97] vs 57% [52 of 91]; *P* = .06). Of all participants, 25% (46 of 188) felt capable of performing CPR because of study participation, 38% (72 of 188) already felt capable before the study, and 4% (8 of 188) felt capable owing to CPR training after the study (*P* = .87). Fifty-five percent of all participants (103 of 188) agreed or strongly agreed with the statement that they would be scared to perform CPR (56% [54 of 97] in the face-to-face group and 54% [49 of 91] in the VR group; *P* = .91).

### Theoretical Knowledge on CPR

Details regarding the individual questions and answers on theoretical CPR knowledge are in Table 3. Overall, 65% of all participants (122 of 188) identified 5 to 6 cm as the correct chest compression depth (face-to-face group, 64 of 97 [66%]; and VR group, 58 of 91 [64%]; *P* = .72) and 57% of all participants (108 of 188) identified the correct chest compression speed of 2 compressions per second (face-to-face group, 52 of 97 [54%]; and VR group, 56 of 91 [62%]; *P* = .54).

### Dissemination of Awareness

#### Action Taken by Participants

In total, 27% of all participants (50 of 188) searched for information on CPR after participating in the trial (28% [27 of 97] in the face-to-face group and 25% [23 of 91] in the VR group; *P* = .24), 15% (29 of 188) underwent instructor-led CPR training after the trial (17% [16 of 97] in the face-to-face group and 14% [13 of 91] in the VR group; *P* = .68), and 27% (50 of 188) indicated that they were planning to undergo CPR training (30% [29 of 97] in the face-to-face group and 23% [21 of 91] in the VR group; *P* = .55) (Table 4).^16^ One percent of all participants (2 of 188) downloaded and/or used the Lifesaver VR application.

#### Action Taken by Family or Acquaintances

Overall, 5% of family member or acquaintances (10 of 188) took concrete action (ie, applied for or participated in a CPR course) (5% [5 of 97] in the face-to-face group and 6% [5 of 91] in the VR group; *P* = .51).

## Discussion

This 6-month follow-up survey of a large randomized clinical trial on short CPR training modules shows that more than 75% of participants are willing to perform CPR if they witness a cardiac arrest and that knowledge retention is high, with a median of 7 of 9 CPR questions answered correctly. In addition, 1 of 6 participants followed an official CPR course after participating in the study. Two-thirds of the respondents reported that they told at least 1 to 10 family members and friends about the importance of CPR. We found no marked differences between participants who received face-to-face training and those who received VR CPR training, although the proportion of participants who were willing to perform CPR was slightly higher in the face-to-face group. Despite these findings, more than 50% of participants reported that they would be scared to perform CPR. These collective findings, as well as a previous report on the quality of CPR,^13^ underscore the potential of short CPR training and indicate that understanding and mitigating the anticipated fear of performing CPR should be investigated further.

### Willingness to Perform CPR

The Lowlands Saves Lives trial demonstrated that high-quality CPR can be achieved by 20-minute CPR training, in line with previous short CPR training modules.^13,17,18^ More participants in the face-to-face group than in the VR group indicated that they would be willing to start CPR; perhaps the lack of instructor and manikin feedback may have left VR-trained participants feeling more uncertain because the reported rates of participants who felt capable to perform CPR tended to be lower in the VR group (Table 2). A previous large randomized clinical trial in which all participants were allowed to train on CPR manikins also demonstrated high willingness to perform CPR.^19^ From an educational perspective, feedback on CPR skills remains an important issue to address in the further development of VR CPR training applications.

The high proportion of participants feeling scared to perform CPR is consistent with previous studies. Sasson et al^12^ demonstrated that fear of doing harm or fear of legal consequences are often-perceived barriers to performing CPR. Our findings indicate that this is an important point to address in future studies, and the most recent guidelines^20^ state that bystander CPR should always be encouraged because the chances of doing harm are outweighed by the potential benefit of CPR.

### Knowledge Retention

Owing to logistic reasons, with attendees living in many places in the Netherlands and abroad, follow-up was focused on theoretical and not practical knowledge retention, which was high and equal among both groups. Previous studies on short CPR training modules also demonstrated high knowledge retention, including practical CPR skills.^17,21^ The fact that CPR skills achieved with short training modules are good and seem retainable underscores their potential because training modalities that allow for easy rehearsal training may appeal to a large target population and may facilitate a more sustained quality of CPR.^22^ This finding is relevant because a decline in practical CPR skills often occurs over time and guidelines stress the importance of frequent retraining to maintain the ability to perform CPR.^8,23^ The importance of frequent retraining is underscored by the low rates of CPR exposure because only 2% of the study participants (3 of 188) witnessed a cardiac arrest nonprofessionally in the 6 months after the study.

### Dissemination of Awareness

Increased bystander willingness to perform CPR may lead to improved outcomes after out-of-hospital cardiac arrest.^3^ An important step toward increasing bystander willingness to perform CPR is creating an awareness of the importance of CPR. To our knowledge, this is the first study to systematically investigate the effect of short CPR training modules on the dissemination of CPR awareness, and the results can be considered promising because the present data indicate that short CPR training modules may increase CPR awareness among participants and their relatives and friends.^24^ After extrapolation, more than 1500 people were informed about the importance of CPR as a result of our event. The fairly high proportion of individuals who participated in a certified 4-hour CPR course after the study and the the fairly high proportion of participants who indicated their willingness to participate in a certified 4-hour CPR course contribute to the effect of our study on the CPR skills of the participants who were recruited at the Lowlands music festival.

### Implications

Together with the previously found high-quality CPR performance,^13,17^ the present findings support using unconventional ways to reach a targeted high-volume, younger population to increase awareness of civilian CPR. The notion of expanding the use of public events for (short) CPR training is supported by the positive evaluation given by participants of CPR training at music festivals in general and of our training modules in particular. A study on regular CPR training assessed public opinion using Twitter and found that the sentiment of most tweets was negative and that time, place, and duration of training were the often-mentioned barriers.^24^

In this context, the short duration of our training modules is another attractive aspect for those unwilling to spend a lot of time learning CPR and maintaining CPR skills. Virtual reality training in particular allows an individual to train whenever and wherever they want. Optimizing ways to maintain CPR skills is a major topic of interest in current CPR guidelines.^8^ As such, comparisons of short VR training with standard CPR training are warranted, particularly for rehearsal training.

Because very few participants downloaded the VR application, future initiatives should focus on the low cost of the application and the potential benefit of sustaining CPR skills and should mention the websites where the application can be downloaded. Because the cost of additional VR equipment was reported to be a main barrier to engaging in follow-up VR training, the existence of low-cost alternatives, such as cardboard VR goggles, should be mentioned. These low-cost alternatives could also be provided for free after study participation to further increase continued at-home training. The application requires further development to improve the achieved compression depth.^13,18^

An important topic for future research is the reported anticipated fear to perform CPR. More in-depth research into underlying causes (eg, the fear of doing harm or underperforming and need for feedback during performance) is needed to address this issue because it may be a barrier to initiating CPR training. In the quest to improve outcomes after cardiac arrest, these topics of future research may help to further improve CPR training and to create more awareness of the importance of CPR training.

### Limitations

This study has some limitations. Not all participants in our study provided informed consent for the follow-up survey, and some did not complete the entire survey, which is a common problem in surveys that may lead to selection bias. In eTable 3 in Supplement 2, we compared participants with and participants without informed consent and found a statistically significantly larger proportion of participants who had witnessed a cardiac arrest among those who provided informed consent. Another factor that may limit generalizability is the fact that we studied young, highly educated festival attendees, some of whom were health care professionals. There was a difference between the proportion of health care professionals and the proportion of participants with an alcohol level of 0.5‰ or higher between the study groups. We performed sensitivity analyses excluding these subgroups (eTables 4 and 5 in Supplement 2), and the overall results and the results regarding knowledge retention were similar to the main results. The main interest of this subanalysis of the randomized clinical trial was not to make a head-to-head comparison but to assess the overall long-term effect of 20-minute CPR training modules during public events. Because we did not perform a survey before the start of the study, we cannot compare pretest and posttest outcome metrics. This issue also applies to the anticipated fear of performing CPR, and our data do not provide insight into the reasons why participants were scared of performing CPR. Data on outcome parameters such as registering for a follow-up course and downloading the application were provided only on posters and folders in the study area, and higher numbers of participants might have registered for a follow-up course and downloaded the application had these factors been addressed during the 20-minute CPR training, using a standardized protocol. Additionally, although we report on high theoretical knowledge retention, our study design did not allow for follow-up on technical skill retention. It is unclear how this theoretical knowledge translates into actual in-field CPR performance.

## Conclusions

In this 6-month follow-up survey of a randomized clinical trial on 20-minute CPR training at a public event, more than three-fourths of young adult participants reported a willingness to perform CPR on a stranger, with slightly higher rates for participants in the face-to-face training group than in the VR training group. Theoretical knowledge retention was good, and dissemination of awareness was high, including 1 of 6 participants partaking in certified CPR training during follow-up. Taken together, these novel CPR training modules are promising and could be further improved by mitigating the fear of performing CPR.

## Notes

Supplement 1.
Trial Protocol

Supplement 2. **eFigure 1.** Graphical Impression of the Study Setting **eFigure 2.** CONSORT Flow Diagram **eTable 1.** Complete Survey **eTable 2.** Baseline Characteristics of Included and Excluded Participants **eTable 3.** Comparison of Participants With and Without Informed Consent for Follow-up **eTable 4.** Summary of Results With Exclusion of All Health Care Providers **eTable 5.** Summary of Results With Exclusion of Participants With Alcohol Levels ≥0.5‰

Supplement 3.
Data Sharing Statement
