# Supplementary material for: Long-term Effect of Face-to-Face vs Virtual Reality Cardiopulmonary Resuscitation (CPR) Training on Willingness to Perform CPR, Retention of Knowledge, and Dissemination of CPR Awareness: A Secondary Analysis of a Randomized Clinical Trial
Source: JAMA Netw Open. 2022 May 19;5(5):e2212964. doi: 10.1001/jamanetworkopen.2022.12964 (PMC9121185; doi:10.1001/jamanetworkopen.2022.12964)
Supplement: Supplement 1. — Trial Protocol [file jamanetwopen-e2212964-s001.pdf]

RESEARCH PROTOCOL

LOWLANDS SAVES LIVES

-

A randomized trial to assess the impact of face-to-face vs. virtual reality training using the Lifesaver VR-app on the quality of cardiopulmonary resuscitation.

PROTOCOL TITLE: A randomized trial to assess the impact of face-to-face vs. virtual reality training using the Lifesaver VR-app on the quality of cardiopulmonary resuscitation.

**Protocol ID**	
**Short title**	**Lowlands saves lives**
**Version**	**1.0**
**Date**	**22-04-2019**
**Project leader**	***Prof. dr. N van Royen, head of department of cardiology, Radboudumc, Nijmegen***
**Principal investigator(s)**	***Dr. MA Brouwer, cardiologist***<br>***J Nas, MSc, study coordinator***<br>***Department of cardiology, Radboudumc, Nijmegen***
**Sponsor**	***Not applicable***
**Subsidising party**	***Not applicable***
**Independent expert (s)**	***Prof. dr. M Edwards***<br>***Department of Trauma Surgery, Radboudumc, Nijmegen***
**Laboratory sites**	***Not applicable***
**Pharmacy**	***Not applicable***

PROTOCOL SIGNATURE SHEET

Name	Signature	Date
**Head of Department:**<br>***Prof. dr. N van Royen***		
**Project leader**<br>***Prof. dr. N van Royen***		
**Study coordinator**<br>***J. Nas, MSc***		

TABLE OF CONTENTS

INTRODUCTION AND RATIONALE 7
1. OBJECTIVES 8
2. STUDY DESIGN 8
3. STUDY POPULATION 10
3.1 Population (base): 10
Adult (≥18 years) Lowlands-attendees. We expect to recruit approximately 300 participants during the 3 full days of festival-attendance. 10
3.2 Inclusion criteria 10
3.3 Exclusion criteria 10
4. INVESTIGATIONAL PRODUCT 10
4.1 Name and description of investigational product(s) 10
4.2 Summary of findings from non-clinical studies 11
4.3 Summary of findings from clinical studies 11
4.4 Summary of known and potential risks and benefits 11
5. METHODS 11
5.1 Study parameters/endpoints 11
5.1.1 Main study parameter/endpoint 11
5.1.2 Secondary study parameters/endpoints (if applicable) 12
5.1.3 Other study parameters (if applicable) 12
5.2 Randomisation, blinding and treatment allocation 12
5.3 Study procedures 12
5.4 Withdrawal of individual subjects 12
5.5 Replacement of individual subjects after withdrawal 13
5.6 Follow-up of subjects withdrawn from treatment 13
5.7 Premature termination of the study 13
6. STATISTICAL ANALYSIS 14
7. ETHICAL CONSIDERATIONS 15
7.1 Regulation statement 15
7.2 Recruitment and consent 15
8. ADMINISTRATIVE ASPECTS, MONITORING AND PUBLICATION 16
8.1 Handling and storage of data and documents 16
8.2 Monitoring and Quality Assurance 16
8.3 Amendments 16
8.4 Annual progress report 16
8.5 Temporary halt and (prematurely) end of study report 16
8.6 Public disclosure and publication policy 16
9. STRUCTURED RISK ANALYSIS 17
9.1 Synthesis 17
10. REFERENCES 18

LIST OF ABBREVIATIONS AND RELEVANT DEFINITIONS

**BLS:**	**Basic life support**
**CPR:**	**Cardiopulmonary resuscitation**
**OHCA:**	**Out-of-hospital cardiac arrest**
**VR:**	**Virtual reality**

SUMMARY

Rationale: In order to optimize survival after out-of-hospital cardiac arrest, basic life support (BLS) training of lay-person volunteers is essential. It is unknown which training method results in the highest quality cardiopulmonary resuscitation (CPR).

Objective: To compare face-to-face CPR training with CPR-training using the Lifesaver virtual reality (VR) app in terms of quality of CPR.

Study design: Randomized controlled trial.

Study population: Adult (≥18 years) Lowlands-festival attendees.

Intervention (if applicable): We will compare face-to-face training with the Lifesaver VR cell phone application, which is an interactive game than can be used for BLS-training. In the latter, users 'resuscitate' a victim of cardiac arrest, while wearing VR-glasses showing a filmed CPR-scenario.

Main study parameters/endpoints: Following the training, participants will perform CPR on a certified CPR-training manikin. Main outcome measure is the mean depth and rate of chest compressions. Secondary outcomes are flow fraction, CPR performance and the proportion of participants with CPR-parameters within guideline range

Nature and extent of the burden and risks associated with participation, benefit and group relatedness: All assessments will be made in one visit of approximately 30 minutes. We will use a questionnaire to assess demographics and previous CPR experience. No follow-up visits are required. No blood samples or other body material will be collected. The benefit for the participants is a basic CPR-lesson.

INTRODUCTION AND RATIONALE

Out-of-hospital cardiac arrest (OHCA) is a major health care problem. [1] In the Netherlands, about 300 OHCAs occur weekly, of which the average chance of survival is 23%. [2] A promising development in the care for cardiac arrest victims is the increased involvement of lay-volunteers in providing cardiopulmonary resuscitation (CPR). High quality bystander CPR while awaiting professional health care providers markedly increases chances of survival. [3, 4]

However, in many OHCAs no bystander CPR is performed.[2] In order to increase these numbers, lay-volunteers should be educated in performing basic life support (BLS). Several training-methods exist, of which face-to-face is the most common.[5] This comprises schooling participants in the basic principles of recognizing cardiac arrest, how to notify the emergency medical services and how to perform CPR. Chest compression training is performed using certified CPR-manikins.

A novel method for CPR-training is the Lifesaver app. Lifesaver (www.life-saver.org.uk) is an innovative, immersive, and interactive game that can be played for free on smartphones, tablets or online. The novel ‘game-in-film’ format provides an engaging learning experience with real life scenarios and users become actively involved with the resuscitation of a victim of cardiac arrest. If a wrong decision is made, the user sees the impact but is then able to rewind and make the correct decision. It was produced by Resuscitation Council (UK) using charitable funds and generates no financial income.

At present, only one study on the Lifesaver app has been conducted.[6] This was a randomized trial, in which it was demonstrated that training using the Lifesaver app can lead to comparable learning outcomes for several key elements of successful CPR. However, that study was conducted before the virtual reality (VR) enhancement of the app. The recently added VR feature allows the users to experience the resuscitation scenario in VR, using specifically designed VR goggles, further enhancing the experience. In this VR setting, users perform chest compressions on a pillow. Furthermore, the previous study was conducted in school children. Thus, data on adults is lacking, but adults are more likely to witness cardiac arrest and be the first on scene in case of OHCA.

Therefore, we aim to perform the first randomized trial comparing CPR quality between face-to-face and Lifesaver VR app CPR training in adults.

1. OBJECTIVES

Primary Objective: To compare CPR quality between face-to-face and Lifesaver VR app CPR-trained adults using a randomized controlled trial.

2. STUDY DESIGN

We will perform a randomized controlled trial. The present study will be performed during the Lowlands-festival (August 16-18, 2019). This is an annual music-festival in the Netherlands with over 50.000 attendees. The present project was selected out of tens of submissions, to be conducted during Lowlands Science, a section of the festival dedicated exclusively to performing scientific research.

All participants will fill-in a questionnaire regarding demographics and previous CPR experience (appendix 1). As the study will be performed on a musical festival where alcohol consumption is common, and alcohol consumption may impact CPR performance, we will also perform an alcohol breathalyzer test. In case of an alcohol level >0.5‰, participants will be asked to perform a tandem gait test. If they are not able to perform this test, participants will be excluded from the study. Participants will be asked if they used any other drugs/narcotics in the 24 hours before participation and will be excluded if they are deemed too intoxicated to participate. Subsequently, the participants will be randomized into either one of the following groups:

1. Face-to-face training. A short face-to-face training by a certified BLS-instructor will be provided.
2. Lifesaver VR app. Participants will be given a VR-headset running the lifesaver app and will go through one complete CPR scenario.

Randomization will be stratified according to alcohol level.

All participants, from both groups, will perform the training under direct supervision of the attending experienced instructors.

Directly following the training, all participants will demonstrate CPR-skills on a certified CPR-training manikin which will register chest compression parameters (depth, rate etc.). Furthermore, a checklist will be scored regarding the required steps for performing adequate CPR (appendix 2). The results of these tests will be registered in an anonymised database. If

a participant provides additional consent, we will make a video recording of the CPR-test. This will allow for independent, external data review.

3. STUDY POPULATION

3.1 Population (base):

Adult (≥18 years) Lowlands-attendees. No minimum number of participants is required. Inclusion will continue for the entire Lowlands festival, regardless of the number of inclusions. We expect to recruit approximately 300 participants during the 3 full days of festival-attendance. The proportion of participants with previous CPR experience is capped at 20%.

3.2 Inclusion criteria

In order to be eligible to participate in this study, a subject must meet all of the following criteria:

1. Adult (≥18 years)
2. Provide informed consent

3.3 Exclusion criteria

A potential subject who meets any of the following criteria will be excluded from participation in this study:

1. Alcohol level >0.5‰ and not able to perform tandem gait test.
2. For any reason not being able to partake in the face-to-face or VR-app training (e.g. clear alcohol or drugs intoxication).
3. For any reason not being able to perform the CPR test on the CPR-manikin (e.g. clear alcohol or drugs intoxication).

4. INVESTIGATIONAL PRODUCT

4.1 Name and description of investigational product(s)

Lifesaver VR app: Lifesaver (www.life-saver.org.uk) is an innovative, immersive, and interactive game that can be played for free on smartphones, tablets or online. The novel ‘game-in-film’ format provides an engaging learning experience with real life scenarios. Users become actively involved with the resuscitation of a victim of cardiac arrest and simulate cardiac compressions by performing compressions on a pillow. If a wrong decision is made, the user sees the impact but is then able to rewind and make the correct decision. The recently added VR feature allows the users to experience the resuscitation scenario in VR, using specifically designed VR goggles, further enhancing

the experience. It was produced by Resuscitation Council (UK) using charitable funds and generates no financial income.

4.2 Summary of findings from non-clinical studies

No non-clinical studies have been performed as these are not applicable to this investigational product.

4.3 Summary of findings from clinical studies

Currently, one study has been performed using the Lifesaver app.[6] In that study, 81 children from UK schools were randomized into a group with face-to-face training only, Lifesaver only or a combination of both. This study demonstrated that the use of Lifesaver by school children, compared to face-to-face training alone, can lead to comparable learning outcomes for several key elements of successful CPR. No adverse events were reported. Data specifically focussing on the Lifesaver VR app is lacking.

4.4 Summary of known and potential risks and benefits

Potential risks: none. The app was specifically designed for lay-persons by the UK Resuscitation Council. There are no known potential adverse events associated with the game. It is increasingly used in UK schools. The game has won several awards, and was nominated for a BAFTA British Academy Award, in the Children's Interactive Category (https://www.elsevier.com/connect/lifesaver-app-teaches-cpr-by-throwing-you-into-the-action). Therefore, we feel that the app is safe to use in Lowlands-attendees.
Potential benefits: acquired basic CPR-skills.

5. METHODS

5.1 Study parameters/endpoints

Study endpoints will be assessed using certified CPR manikins and by assessors that are blinded for the study intervention. A sub-set of the CPR-tests will be reviewed by external, independent assessors. For this we will use video-recordings, for which we will ask additional informed consent.

5.1.1 Main study parameter/endpoint

Chest compression quality, measured as mean chest compression rate (compressions per minute) and depth (mm) using a certified CPR-manikin.

5.1.2 Secondary study parameters/endpoints (if applicable)

CPR-score as measured by the CPR-checklist (appendix 2) and flow fraction (percentage of time where compressions given) measured using a certified CPR-manikin. CPR-parameters within guideline range, as a binary variable.

5.1.3 Other study parameters (if applicable)

We will collect data on age, sex, weight, educational level, previous CPR experience and training, use of drugs, and alcohol intake using a breathalyzer alcohol test.

5.2 Randomisation, blinding and treatment allocation

Participants will be randomized into one of the two groups using the online CASTOR data management system. Due to the nature of the intervention, no participant blinding will be performed. CPR-quality will be measured by certified CPR-manikins and assessors blinder for study group.

5.3 Study procedures

Participants will undergo the following procedures:

1. Filling in a questionnaire on demographics, drug use and previous CPR experience and/or training
2. Alcohol breathalyzer test.
3. CPR-training using one of the following two methods: face-to-face training, or training using the Lifesaver VR app.
4. CPR quality test, to be performed on a certified CPR manikin, under supervision of one of the assessors. The assessor will also measure the CPR-score using the CPR-checklist of the European Resuscitation Council course assessments documents (appendix 2). If a participant provides additional consent, we will make a video recording of the CPR-test. A random sample of the exams will be reviewed by an external, independent event committee.

We will also ask the participants if they would like to be approached for a follow-up questionnaire on this subject. This is voluntarily and not obligatory to participate in this study.

5.4 Withdrawal of individual subjects

Subjects can discontinue the study at any time for any reason if they wish to do so without any consequences.

5.5 Replacement of individual subjects after withdrawal

Not applicable.

5.6 Follow-up of subjects withdrawn from treatment

No follow-up will be performed in participants withdrawn from the study.

5.7 Premature termination of the study

If a participant is unable to complete the CPR-training or CPR-test for any reason, the participant may be withdrawn from the study. The data gathered until that point will be used for analyses.

6. STATISTICAL ANALYSIS

Continuous variables will be assessed for normal distribution and reported as means (standard deviation) or medians (interquartile range), whichever appropriate. Continuous data will be compared using a student's T-test or Mann-Whitney U test, whichever appropriate. Categorical variables will be reported as numbers (%) and compared using chi-squared or pearson exact tests, whichever appropriate.
All baseline variables (demographics, previous CPR experience) and outcome data (CPR rate and depth, CPR-score, flow fraction) variables will also be compared between the two study groups using the abovementioned tests. In case of confounding variables, we will correct the comparisons on the outcome measures between the study groups for these confounders using Analysis of Covariance (ANCOVA).
A p-value of <0.05 will be considered statistically significant. Analyses will be performed using SPSS (IBM SPSS version 25, IBM Corp., Armonk, NY, USA).

7. ETHICAL CONSIDERATIONS

7.1 Regulation statement

The study will be conducted according to the principles of the Declaration of Helsinki (most recent version established at the 64th WMA General Assembly, Fortaleza, Brazil, October 2013) and in accordance with the Medical Research Involving Human Subjects Act (WMO).

7.2 Recruitment and consent

All participants will be recruited during the Lowlands festival (August 16-18, 2019). In case of interest in participating in the study, Lowlands attendees can report to our booth in the Lowlands Science area. They will then be informed by one of the present research physicians, trained in the study protocol. The participant will receive a participant information letter and informed consent form. Subjects have until the end of the festival to consider their decision and can report back to the booth in case they are willing to participate. If at any point during the study, participants feel uncomfortable with the CPR-lessons (face-to-face or VR), they can stop participating in the study. To allow for external data review, we will ask for additional consent to make a video-recording of the CPR-test. This is voluntarily and not obligatory to participate in the main study.

We will also ask the participants if they would like to be approached for a follow-up questionnaire on this subject. This is also voluntarily and not obligatory to participate in the main study.

The participant information letter is provided in appendix 3. Participants will be send an electronic version of the participant information folder if they want to.

8. ADMINISTRATIVE ASPECTS, MONITORING AND PUBLICATION

8.1 Handling and storage of data and documents

When a participant signed for informed consent, a case report form (CRF) number will be generated and all further documents will be coded with this CRF number, which precludes directly relating data to individuals. The transcription key is only available to the project leader and study coordinator and will be protected by a password. All data will be entered into the eCRF using the Castor database program under their unique identification number. There is no risk of incidental findings. Data will be stored on a secured location for 15 years. The handling of personal data complies with the EU General Data Protection Regulation and the Dutch Act on Implementation of the General Data Protection Regulation.

8.2 Monitoring and Quality Assurance

Data will be entered in Castor, which has been officially approved for study purposes.

8.3 Amendments

Amendments are changes made to the research after a favourable opinion by the accredited METC has been given. All amendments will be notified to this METC.

8.4 Annual progress report

Due to the short duration of the study, no annual progress report will be submitted.

8.5 Temporary halt and (prematurely) end of study report

The investigator/sponsor will notify the accredited METC of the end of the study within a period of 8 weeks. The end of the study is defined as the last participant's last visit.
The sponsor will notify the METC immediately of a temporary halt of the study, including the reason of such an action. In case the study is ended prematurely, the sponsor will notify the accredited METC within 15 days, including the reasons for the premature termination. Within one year after the end of the study, the investigator/sponsor will submit a final study report with the results of the study, including any publications/abstracts of the study, to the accredited METC.

8.6 Public disclosure and publication policy

The study coordinator and project leader will ensure publication of the data, in close collaboration with all co-workers in this study.

9. STRUCTURED RISK ANALYSIS

9.1 Synthesis

We will use the Lifesaver VR app for its designated purpose: to educate lay-persons in performing CPR. Previous studies have not indicated any potential risks, and demonstrated the feasibility of the product for this purpose. The addition of the VR functionality is unlikely to alter the risk profile of the app. All training (face-to-face and VR-training) will be performed under direct supervision of experienced physicians. No additional procedures are undertaken. Therefore, we feel that participants have no risk of harm or other adverse events.

10. REFERENCES

[1] Atwood C, Eisenberg MS, Herlitz J, Rea TD. Incidence of EMS-treated out-of-hospital cardiac arrest in Europe. Resuscitation 2005;67:75-80.

[2] Zijlstra JA, Radstok A, Pijls R, et al. Overleving na een reanimatie buiten het ziekenhuis: vergelijking van de resultaten van 6 verschillende Nederlands regio's. In: Reanimatie in Nederland 2016. Den Haag: Hartsstichting 2016.

[3] Malta Hansen C, Kragholm K, Pearson DA, et al. Association of Bystander and First-Responder Intervention With Survival After Out-of-Hospital Cardiac Arrest in North Carolina, 2010-2013. Jama 2015;314:255-64.

[4] Nas J, Thannhauser J, Herrmann JJ, et al. Changes in automated external defibrillator use and survival after out-of-hospital cardiac arrest in the Nijmegen area. Neth Heart J 2018;26:600-05.

[5] Perkins GD, Handley AJ, Koster RW, et al. European Resuscitation Council Guidelines for Resuscitation 2015: Section 2. Adult basic life support and automated external defibrillation. Resuscitation 2015;95:81-99.

[6] Yeung J, Kovic I, Vidacic M, et al. The school Lifesavers study-A randomised controlled trial comparing the impact of Lifesaver only, face-to-face training only, and Lifesaver with face-to-face training on CPR knowledge, skills and attitudes in UK school children. Resuscitation 2017;120:138-45.

APPENDIX 1 QUESTIONNAIRES

Vragenlijst voor proefpersonen (Nederlands)

Studie nummer & instructeur<br>(in te vullen door onderzoeker)		
E-mail adres *		
Geslacht	O Vrouw<br>O Man<br>O Wenst deze informatie niet te delen	
Leeftijd		
Gewicht		
Hoogst genoten opleiding	O Basisonderwijs<br>O Middelbaar onderwijs<br>O MBO<br>O HBO<br>O WO	
Beroep in de zorg	O Ja<br>O Nee	
Heeft u, buiten alcohol, de afgelopen 24 uur verdovende middelen gebruikt?	O Ja, welke:<br>O Nee<br>O Wenst deze informatie niet te delen	
Ervaring met reanimatie		
Reanimatie cursus	O Nee<br>O Ja, welk niveau:<br>O De Lifesaver app<br>O BLS<br>O BLS+AED<br>O ALS<br>O Anders, namelijk:<br><br>Datum laatste cursus:	
Reanimatie meegemaakt	O Nee<br>O Ja, … keer<br>O Enkel ooggetuige<br>O Mee gereanimeerd als leek<br>O Mee gereanimeerd als professional	
Het slachtoffer was een<br>(kies een of beide)	O Onbekende<br>O Familielid of bekend	

* Als u benaderd wil worden voor een vervolg vragenlijst of als u de informatie digitaal wil ontvangen.

Questionnaire for study participants (English)

Study number & instructor<br>(to be completed by investigator)		
E-mail address*		
Sex	O Female<br>O Male<br>O Do not wish to disclose	
Age		
Weight		
Highest level of education	O Primary education<br>O Secondary education<br>O Short cycle tertiary education<br>O Bachelor or equivalent<br>O Master or equivalent	
Healthcare professional	O Yes<br>O No	
Have you used any drugs/narcotics in the past 24 hours, besides alcohol?	O Yes, which:<br>O No<br>O Do not wish to disclose	
Previous CPR experience		
CPR course	O No<br>O Yes, which level<br>O BLS<br>O BLS+AED<br>O ALS<br>O Other:<br><br>Date last course:	
Witnessed a cardiac arrest	O No<br>O Yes, ... times<br>O Only as a witness<br>O CPR performed as bystander/lay-person<br>O CPR performed as healthcare professional	
The victim was	O A stranger<br>O A relative or other acquaintance	

* If you want to be approached for a follow-up study or receive the information digitally

APPENDIX 2 CPR SKILL ASSESSMENT TEST

(European Resuscitation Council CPR/AED course assessment document)

EUROPEAN RESUSCITATION COUNCIL

2016.V2

BLS assessment record

Candidate Name: Date: Instructor:

Skill	The candidate	Achieved		Comments
		Yes	No	
Check response	Demonstrates gently shaking and shouting to establish responsiveness			
Assess breathing	Demonstrates head tilt and chin lift			
Assess breathing	Demonstrates look, listen and feel for normal breathing for no more than 10 sec (does not count aloud)			
Call emergency services (Get help)	Describes how to phone for emergency services: 112, unresponsive and non-breathing victim, AED			
Chest compressions	Demonstrates effective chest compressions; rate 100-120/min, depth 5-6 cm; hand position: centre of the chest. Minimises interruptions in chest compressions			
Rescue breaths	Demonstrates rescue breaths sufficient to cause the chest to rise and fall			
Compression : ventilation ratio	Demonstrates ratio of 30 compressions to 2 ventilations			
Activate AED	Switch the AED on or, if a helper is present, ask him/her to do it			
Attach pads	Demonstrates attaching pads in correct position			
Stand clear	Allows rhythm analysis whilst making sure that nobody touches the victim (including visual sweep and verbal instruction)			
Deliver shock	Demonstrates rapid and safe delivery of a shock (including visual sweep and verbal instruction to stand clear)			
Follow AED instructions	Demonstrates listening to and executing AED instructions			
CPR	Minimises interruptions in chest compressions and demonstrates correct sequence in ratio of 30 compressions to 2 ventilations			

APPENDIX 3a PARTICIPANT INFORMATION FOLDER (DUTCH)

Kwaliteit van reanimatie na training door een instructeur vergeleken met training met een virtual reality app

(Lowlands saves Lives - A randomized trial to assess the impact of face-to-face vs. virtual reality training using the Lifesaver VR-app on the quality of cardiopulmonary resuscitation)

Inleiding

Wij vragen u om mee te doen aan een medisch-wetenschappelijk onderzoek. Meedoen is vrijwillig. Om mee te doen is wel uw schriftelijke toestemming nodig. Voordat u beslist of u wilt meedoen aan dit onderzoek, krijgt u uitleg over wat het onderzoek inhoudt. Lees deze informatie rustig door en vraag de onderzoeker uitleg als u vragen heeft.

1. Algemene informatie en doel van het onderzoek

Wekelijks krijgen er in Nederland 300 mensen een hartstilstand. De gemiddelde overlevingskans is 23%. Die kans wordt groter als er wordt gestart met reanimeren voordat de ambulance aanwezig is. Helaas gebeurt dit niet altijd, omdat de omstanders vaak niet kunnen reanimeren. Om dit probleem aan te pakken, is het nodig om meer mensen te leren reanimeren. Dit kan op verschillende manieren. De meest gangbare manier is door een reanimatietraining te volgen bij een instructeur. Een andere manier is door gebruik te maken van de smartphone-app “Lifesaver”. Dit is een app die speciaal is ontwikkeld om te leren reanimeren. Het is onbekend welke manier van les geven leidt tot de beste kwaliteit van reanimatie. Daarom hebben we dit onderzoek opgezet.

Wij willen onderzoeken welke vorm van reanimatietraining leidt tot de beste kwaliteit van reanimatie: les door een instructeur, of les met een reanimatie-app. Dit onderzoek wordt uitgevoerd tijdens het Lowlands-festival en deelname duurt ongeveer 15 minuten. We verwachten 300 deelnemers. Dit onderzoek is beoordeeld door ethische adviescommissie van het Radboudumc, Nijmegen.

2. Wat meedoen inhoudt

Als u meedoet vragen we u om een korte vragenlijst in te vullen. Hierop vragen we onder andere naar uw leeftijd en gewicht en naar voorgaande ervaring met reanimatie. Omdat het gebruik van alcohol de reanimatiekwaliteit kan beïnvloeden, willen we uw alcoholpromillage meten met een alcohol ademtest. Als het alcoholpromillage >0.5‰ is, vragen we u om over een touwtje te lopen om te testen of u in staat bent om te reanimeren. Ook vragen we naar drugsgebruik in de afgelopen 24 uur. Vervolgens wordt u door middel van een loting ingedeeld in een van de twee groepen:

1. Reanimatieles door een instructeur.
2. Reanimatieles door middel van de Lifesaver virtual reality app, waarbij u een realistisch reanimatie-scenario doorloopt met een virtual reality bril.

Na het doorlopen van de reanimatieles legt u een reanimatietest af. De test bestaat uit een korte reanimatie op een speciale reanimatiepop. De pop registreert de kwaliteit van de reanimatie. Een onderzoeker houdt ook een score bij door middel van een checklist. Indien u daar apart toestemming

voor geeft, zullen we het examen opnemen op video. Daardoor kan een externe beoordelaar het examen nog eens nakijken. Hierna is het onderzoek afgelopen. Er zijn geen risico's verbonden aan deelname. U krijgt voor deelname geen officieel reanimatiecertificaat, omdat de tijd te kort is om een hele cursus te verzorgen. Als u wil deelnemen aan een vervolgvragenlijst, kunt u uw e-mail adres achterlaten. Dit is optioneel.

3. Als u niet wilt meedoen en/of wilt stoppen met het onderzoek

U beslist zelf of u meedoet aan het onderzoek, deelname is vrijwillig. U kunt op elk moment stoppen met deelname aan het onderzoek. Uw deelname stopt als u zelf kiest om te stoppen of als alle metingen gedaan zijn. Het hele onderzoek is afgelopen als alle deelnemers klaar zijn.

4. Gebruik en bewaren van uw gegevens

Voor dit onderzoek worden uw persoonsgegevens verzameld, gebruikt en bewaard. Het gaat om gegevens zoals uw leeftijd, gewicht en, indien u benaderd wil worden voor vervolgonderzoek, uw e-mailadres. Indien u daar apart toestemming voor geeft, maken we video-opnames van de reanimatietest. Het verzamelen, gebruiken en bewaren van uw gegevens is nodig om de vragen die in dit onderzoek worden gesteld te kunnen beantwoorden en de resultaten te kunnen publiceren. Wij vragen voor het gebruik van uw gegevens uw toestemming. Een uitgebreide tekst over het gebruiken en bewaren van uw gegevens vindt u in Bijlage 1. Dit onderzoek voldoet aan de Algemene verordening gegevensbescherming (AVG)

5. Heeft u vragen?

Bij vragen kunt u contact opnemen met het onderzoeksteam. De hoofdonderzoekers zijn J Nas en dr. MA Brouwer. Zij zijn aanwezig op het Lowlands festival en telefonisch bereikbaar via 024-3616785 (buiten kantooruren op 0621195438). De onafhankelijke arts voor dit onderzoek is prof. dr. Michael Edwards, traumachirurg, bereikbaar op 024-3613871. Bij problemen of klachten met betrekking tot dit onderzoek, die u niet met het onderzoeksteam kunt bespreken, kunt u contact opnemen met de Klachtencommissie van het Radboudumc, op telefoonnummer: 024-3613191.

6. Ondertekening toestemmingsformulier

Wanneer u voldoende bedenktijd heeft gehad, wordt u gevraagd te beslissen over deelname aan dit onderzoek. Indien u toestemming geeft, vragen wij u dat op de bijbehorende toestemmingsverklaring schriftelijk te bevestigen. Door uw schriftelijke toestemming geeft u aan dat u de informatie heeft begrepen en instemt met deelname aan het onderzoek. Het handtekeningenblad wordt door de onderzoeker bewaard. Zowel uzelf als de onderzoeker ontvangen een getekende versie van deze toestemmingsverklaring.

Bijlage 1: Gebruik en bewaren van uw gegevens

Kwaliteit van reanimatie na training door een instructeur vergeleken met training met een virtual reality app

Voor dit onderzoek worden uw persoonsgegevens verzameld, gebruikt en bewaard. Het gaat om gegevens zoals uw geboortedatum, e-mail adres en om gegevens over uw gezondheid. Het verzamelen, gebruiken en bewaren van uw gegevens is nodig om de vragen die in dit onderzoek worden gesteld te kunnen beantwoorden en de resultaten te kunnen publiceren. Wij vragen voor het gebruik van uw gegevens.

Vertrouwelijkheid van uw gegevens
Om uw privacy te beschermen krijgen uw gegevens een code. Uw e-mail adres en andere gegevens die u direct kunnen identificeren worden daarbij weggelaten. Alleen met de sleutel van de code zijn gegevens tot u te herleiden. De sleutel van de code blijft veilig opgeborgen in de lokale onderzoeksinstelling. De gegevens die naar de opdrachtgever worden gestuurd bevatten alleen de code, maar niet uw naam of andere gegevens waarmee u kunt worden geïdentificeerd. Ook in rapporten en publicaties over het onderzoek zijn de gegevens niet tot u te herleiden.

Toegang tot uw gegevens voor controle
Sommige personen kunnen op de onderzoekslocatie toegang krijgen tot al uw gegevens. Ook tot de gegevens zonder code. Dit is nodig om te kunnen controleren of het onderzoek goed en betrouwbaar is uitgevoerd. Personen die ter controle inzage krijgen in uw gegevens zijn het team van Lowlands Saves Lives, een externe instantie die de gegevens controleert en nationale en toezichthoudende autoriteiten, bijvoorbeeld de Inspectie Gezondheidszorg en Jeugd. De video-opnames worden door externe beoordelaars bekeken. Hierna worden de video-opnames bewaard op een beveiligde locatie. Alle betrokken partijen houden uw gegevens geheim. Wij vragen u voor deze inzage toestemming te geven.

Bewaartermijn gegevens
Uw gegevens moeten 15 jaar worden bewaard op de onderzoekslocatie.

Intrekken toestemming
U kunt uw toestemming voor gebruik van uw persoonsgegevens altijd weer intrekken. De onderzoeksgegevens die zijn verzameld tot het moment dat u uw toestemming intrekt worden nog wel gebruikt in het onderzoek.

Meer informatie over uw rechten bij verwerking van gegevens
Voor algemene informatie over uw rechten bij verwerking van uw persoonsgegevens kunt u de website van de Autoriteit Persoonsgegevens raadplegen. Bij vragen over uw rechten kunt u contact opnemen met de verantwoordelijke voor de verwerking van uw persoonsgegevens. Voor dit onderzoek is dat het Radboudumc, afdeling cardiologie. Telefoonnummer 024-3616785.

Bij vragen of klachten over de verwerking van uw persoonsgegevens raden we u aan eerst contact op te nemen met de onderzoekslocatie. U kunt ook contact opnemen met de Functionaris voor de Gegevensbescherming van het Radboudumc (gegevensbescherming@radboudumc.nl) of de Autoriteit Persoonsgegevens.

Bijlage 2: toestemmingsformulier deelnemer

Kwaliteit van reanimatie na training door een instructeur vergeleken met training met een virtual reality app

- Ik heb de informatiebrief gelezen. Ook kon ik vragen stellen. Mijn vragen zijn voldoende beantwoord. Ik had genoeg tijd om te beslissen of ik meedoe.
- Ik weet dat meedoen vrijwillig is. Ook weet ik dat ik op ieder moment kan beslissen om toch niet mee te doen of te stoppen met het onderzoek. Daarvoor hoef ik geen reden te geven.
- Ik geef toestemming voor het verzamelen en gebruiken van mijn gegevens voor de beantwoording van de onderzoeksvraag in dit onderzoek.
- Ik weet dat voor de controle van het onderzoek sommige mensen toegang tot al mijn gegevens kunnen krijgen. Die mensen staan vermeld in deze informatiebrief. Ik geef toestemming voor die inzage door deze personen.
- Ik geef **O wel**

  O geen toestemming om mij na dit onderzoek te benaderen voor een vervolg vragenlijst over dit onderwerp

- Ik geef **O wel**

  O geen toestemming om beelden van de reanimatie-test op te nemen met een videorecorder voor externe beoordeling

Ik wil meedoen aan dit onderzoek.

Naam deelnemer:

Handtekening: Datum : __ / __ / __

Ik verklaar dat ik deze deelnemer volledig heb geïnformeerd over het genoemde onderzoek.

Als er tijdens het onderzoek informatie bekend wordt die de toestemming van de deelnemer zou kunnen beïnvloeden, dan breng ik hem/haar daarvan tijdig op de hoogte.

Naam onderzoeker (of diens vertegenwoordiger):

Handtekening: Datum: __ / __ / __

Quality of resuscitation after training by an instructor compared to training with a virtual reality app

(Lowlands saves Lives: A randomized trial to assess the impact of face-to-face vs. virtual reality training using the Lifesaver VR-app on the quality of cardiopulmonary resuscitation.)

Introduction

Dear sir/madam,

We ask you to participate in a medical-scientific study. Participation is voluntary. Participation requires your written consent. Before you decide whether you want to participate in this study, you will be given an explanation about what the study involves. Please read this information carefully and ask the investigator for an explanation if you have any questions.

1. General information and purpose of the study

Weekly, 300 persons experience a cardiac arrest in the Netherlands. The average chances of survival are 23%. This chance increases if cardiopulmonary resuscitation (CPR) is initiated before ambulance arrival. Unfortunately, this not always happens, as bystanders often do not know how to perform CPR. To address this issue, it is necessary to educate more people in performing CPR. This can be done in several ways. The most common way is by attending a course with an instructor. Another way is to use the smartphone-app “Lifesaver”. This is an app that has been specifically developed to learn how to perform CPR. It is unknown which form of training results in the highest quality CPR. Therefore, we initiated this study.

We want to investigate which form of CPR-training leads to the highest quality CPR: CPR-training by an instructor, or CPR-training using a CPR-app. This study will be performed during the Lowlands-festival and participation will take about 15 minutes. We expect 300 participants. The Medical Research Ethics Committee of the Radboudumc has approved this study.

2. What participation involves

If you participate in this study, you are asked to fill in a short questionnaire. This comprises questions about your age and weight and previous CPR experience. As the use of alcohol may impact the quality of CPR, we want to measure your alcohol level using a breathalyzer test. If the alcohol level is >0.5‰, you will be asked to walk over a rope to test if you are able to perform CPR. We will also ask you about any drug use in the past 24 hours. Subsequently, you will be randomly allocated to one of the following two groups:

1. CPR-training by an instructor
2. CPR-training using the Lifesaver virtual reality app, in which you will go through a realistic CPR-scenario using virtual reality goggles.

After completing the CPR-training you will take a CPR-test. This test consists of shortly performing CPR on a designated resuscitation-manikin. This manikin will register the quality of the chest

compressions. A researcher will also keep score of your performance using a checklist. If you give separate permission, we will make a video recording of the post-training test. This will allow an external assessor to review the exam. After this test, the study is completed. There are no follow-up measurements. There are no risks involved in your participation. You will not receive an official CPR-certificate, because the time is too short to provide a complete CPR-course. If you want to participate in a follow-up questionnaire, you can leave your e-mail address. This is optional.

3. If you do not want to participate and/or stop participating in the study

You decide whether or not to participate in the study. Participation is voluntary. You can decide to stop participating in the study at any point. Your participation in the study ends if you choose to stop or if all measurements have been completed. The study is concluded once all the participants have completed the study.

5. Usage and storage of your data

Your personal data will be collected, used and stored for this study. This concerns data such as your age, weight and, in case you want to participate in a follow-up study, your e-mail address. If you give separate permission, we will make video-recordings of the CPR-test. The collection, use and storage of your data is required to answer the questions asked in this study and to publish the results. We ask your permission for the use of your data. An extensive text on the usage and storage of your data can be found in Appendix 1. This study complies with the General Data Protection Regulation.

6. Do you have any questions?

In case of any questions you can contact the study team. The principal investigators are J Nas and dr. MA Brouwer. Both researchers are present at the Lowlands festival and can be reached by phone on 024-3616785 (outside office hours on 0621195438). The independent expert is prof. dr. Michael Edwards, trauma surgeon, who can be reached on 024-3613871. In case of any problems or complaints regarding the study, that you can not discuss with the study team, you can contact the Complaints Committee of the Radboudumc (024-3613191)

7. Signing the consent form

When you have had sufficient time for reflection, you will be asked to decide on participation in this study. If you give permission, we will ask you to confirm this in writing on the appended consent form. By your written permission you indicate that you have understood the information and consent to participation in the study. Yourself and the investigator will receive a signed copy of the consent form.

Appendix 1: Usage and storage of your data

Quality of resuscitation after training by an instructor compared to training with a virtual reality app

Your personal will be collected, used and stored for this study. This concerns data such as your age, weight and, in case you want to participate in a follow-up study, your e-mail address. The collection, use and storage of your is required to answer the questions asked in this study and to publish the results. We ask your permission for the use of your data.

Confidentiality of your data
To protect your privacy, your data will be given a code. Your name and other information that can directly identify you, will be omitted. Data can only be traced back to you with the encryption key. The encryption key remains safely stored in the local research institute. The data that will be used for analyses will only contain the code, not your name or other data with which you can be identified. The data cannot be traced back to you in reports and publications about the study.

Access to your data for verification
Some people can access all your data at the research location. Including the data without a code. This is necessary to check whether the study is being conducted in a good and reliable manner. Persons who have access to your data for review are the team of Lowlands Saves Lives, an external party that checks the data and national authorities, for example, the Healthcare and Youth Inspectorate. The video recordings will be review by external assessors. Afterwards, the recordings will be stored on a safe location. All parties involved will keep your data confidential. We ask you to consent to this access.

Retention period of your data
Your data must be kept for 15 years at the research location.

Withdrawing consent
You can withdraw your consent to the use of your personal data at any time. The study data collected until the moment you withdraw your consent will still be used in the study.

More information about your rights when processing data
For general information about your rights when processing your personal data, you can consult the website of the Dutch Data Protection Authority. If you have questions about your rights, please contact the person responsible for the processing of your personal data. For this study, that is the Radboudumc, department of cardiology. Telephone number 024-3616785.

If you have questions or complaints about the processing of your personal data, we advise you to first contact the research location. You can also contact the Data Protection Officer of the institution (gegevensbescherming@radboudumc.nl) or the Dutch Data Protection Authority.

Appendix 2: Subject Consent Form

Quality of resuscitation after training by an instructor compared to training with a virtual reality app

- I have read the subject information form. I was also able to ask questions. My questions have been answered to my satisfaction. I had enough time to decide whether to participate.
- I know that participation is voluntary. I know that I may decide at any time not to participate after all or to withdraw from the study. I do not need to give a reason for this.
- I give permission for the collection and use of my data to answer the research question in this study.
- I know that some people may have access to all my data to verify the study. These people are listed in this information sheet. I consent to the inspection by them.
- I **O do**
  O do not give consent to approach me for a follow-up questionnaire on this subject

- I **O do**
  O do not give consent to make a video-recording of the CPR-test for external review

- I want to participate in this study

Name of study subject:

Signature: Date: __ / __ / __

I hereby declare that I have fully informed this study subject about this study.

If information comes to light during the course of the study that could affect the study subject's consent, I will inform him/her of this in a timely fashion.

Name of investigator (or his/her representative):

Signature: Date:__ / __ / __
