# Supplementary material for: Long-term Effect of Face-to-Face vs Virtual Reality Cardiopulmonary Resuscitation (CPR) Training on Willingness to Perform CPR, Retention of Knowledge, and Dissemination of CPR Awareness: A Secondary Analysis of a Randomized Clinical Trial
Source: JAMA Netw Open. 2022 May 19;5(5):e2212964. doi: 10.1001/jamanetworkopen.2022.12964 (PMC9121185; doi:10.1001/jamanetworkopen.2022.12964)
Supplement: Supplement 2. — eFigure 1. Graphical Impression of the Study Setting eFigure 2. CONSORT Flow Diagram eTable 1. Complete Survey eTable 2. Baseline Characteristics of Included and Excluded Participants eTable 3. Comparison of Participants With and Without Informed Consent for Follow-up eTable 4. Summary of Results With Exclusion of All Health Care Providers eTable 5. Summary of Results With Exclusion of Participants With Alcohol Levels ≥0.5‰ [file jamanetwopen-e2212964-s002.pdf]

Supplementary Online Content

Nas J, Thannhauser J, Konijnenberg LSF, et al. Long-term effect of face-to-face vs virtual reality cardiopulmonary resuscitation (CPR) training on willingness to perform CPR, retention of knowledge, and dissemination of CPR awareness: a secondary analysis of a randomized clinical trial. *JAMA Netw Open.* 2022;5(5):e2212964. doi:10.1001/jamanetworkopen.2022.12964

eFigure 1. Graphical Impression of the Study Setting
eFigure 2. CONSORT Flow Diagram
eTable 1. Complete Survey
eTable 2. Baseline Characteristics of Included and Excluded Participants
eTable 3. Comparison of Participants With and Without Informed Consent for Follow-up
eTable 4. Summary of Results With Exclusion of All Health Care Providers
eTable 5. Summary of Results With Exclusion of Participants With Alcohol Levels ≥0.5‰

This supplementary material has been provided by the authors to give readers additional information about their work.

© 2022 Nas J et al. *JAMA Network Open.*

eFigure 1. Graphical Impression of the Study Setting

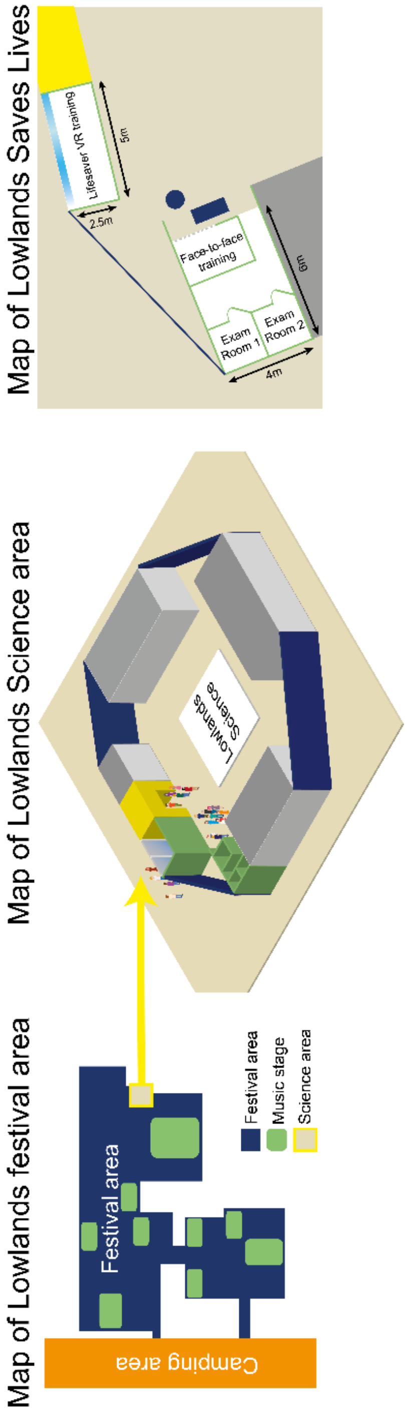

Schematic representation of the study setting. Lowlands Science was an area on the Lowlands festival that was separated from the rest of the festival. Several studies were conducted on Lowlands Science. The square footage of the area for study conduction was pre-specified and could not be expanded.

© 2022 Nas J et al. *JAMA Network Open.*

eFigure 2. CONSORT Flow Diagram

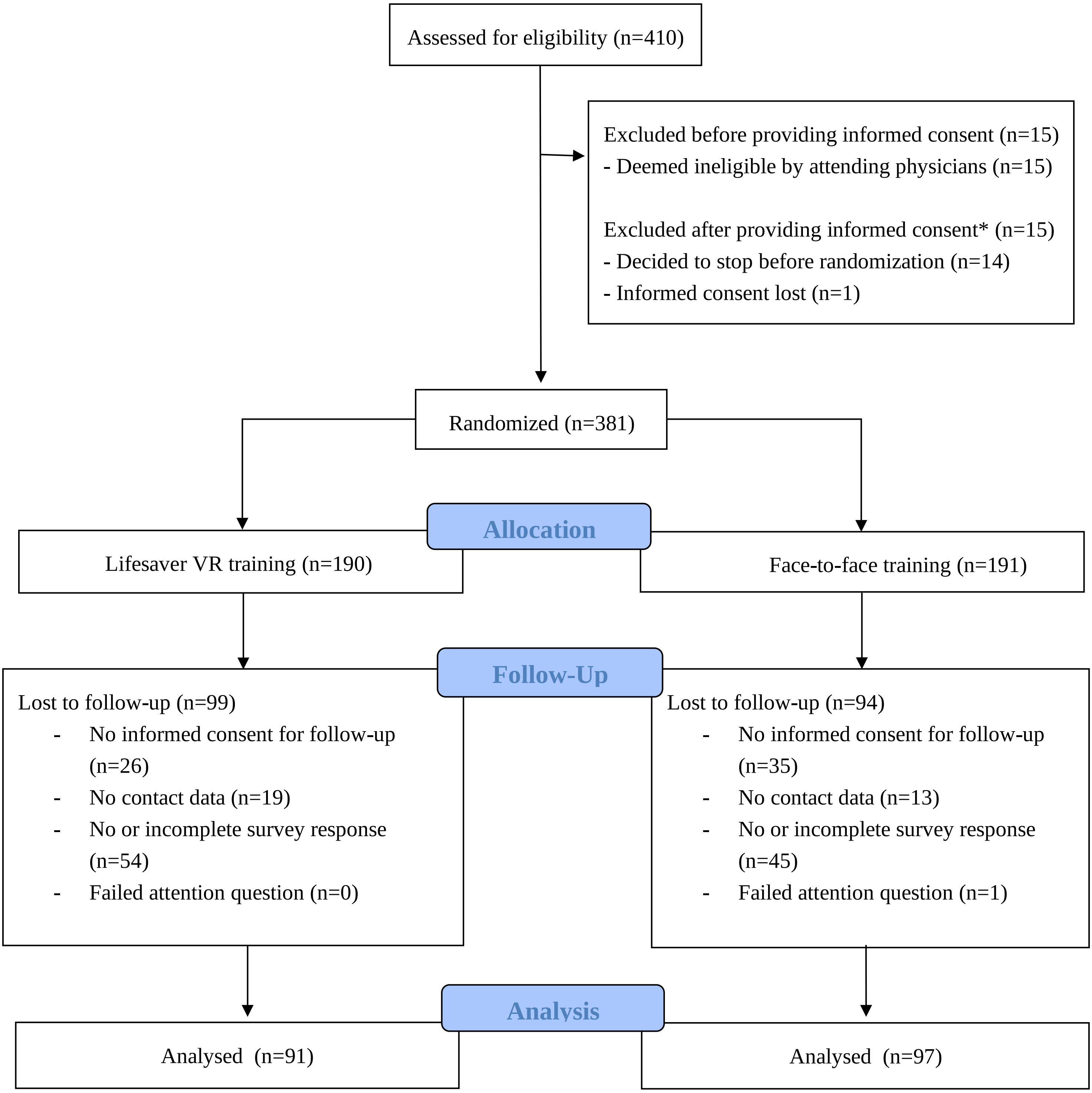

** All included participants were able to pass the tandem gait test, if applicable. VR = Virtual reality.*
CPR = Cardiopulmonary resuscitation.

© 2022 Nas J et al. *JAMA Network Open.*

eTable 1. Complete Survey

**Question**	**Answer choices**	**Question type***
Which training did you follow?	• Face-to-face training<br>• Lifesaver Virtual Reality training	Attention question
**General impression of the study**		
How would you rate your overall experience with the study?	• Positive<br>• Fairly positive<br>• Neutral<br>• Fairly negative<br>• Negative	Informational question
How would you rate the training that you followed?	• Positive<br>• Fairly positive<br>• Neutral<br>• Fairly negative<br>• Negative	Informational question
How do you feel about receiving CPR-training on a music festival?	• Positive<br>• Fairly positive<br>• Neutral<br>• Fairly negative<br>• Negative	Informational question
**Self-reported attitude and willingness**		
Do you feel capable to perform CPR following study participation?	• Yes, because of study participation<br>• Yes, already before the study<br>• Yes, because I did a CPR-training after the study<br>• No	Informational question
If you were to witness a cardiac arrest of an unknown person, would you start CPR?	• Yes<br>• No<br>• Don't know	Informational question
If you were to witness a cardiac arrest of a family member or friend, would you start CPR?	• Yes<br>• No<br>• Don't know	Informational question
I have the feeling that I am capable of helping somebody with cardiac arrest	• Strongly disagree<br>• Disagree<br>• Neutral<br>• Agree<br>• Strongly agree	Informational question
I would be scared to perform CPR	• Strongly disagree<br>• Disagree<br>• Neutral<br>• Agree<br>• Strongly agree	Informational question
I don't want to perform mouth-to-mouth on a stranger	• Strongly disagree<br>• Disagree<br>• Neutral<br>• Agree<br>• Strongly agree	Informational question
**Action taken after study participation - self**		
After participating in the study, did you look for more information on CPR, for example online?	• Don't know<br>• No<br>• Yes<br>▪ On CPR in general<br>▪ On Lifesaver VR<br>▪ Apply for CPR-course<br>▪ Become a registered volunteer<br>▪ Where the nearest AED is<br>▪ Other	Informational question
Did you follow an instructor-led CPR-training following study participation?	• Yes<br>• No	Informational question

© 2022 Nas J et al. *JAMA Network Open.*

	▪ I feel capable enough<br>▪ It costs too much time<br>▪ It is too expensive<br>▪ Not yet, but planning to<br>▪ Other	
Did you download the Lifesaver app following study participation?	• Yes, downloaded and planning to train<br>• Yes, also trained<br>• No<br>▪ Planning to<br>▪ I feel capable enough<br>▪ Don't know where to find it<br>▪ Costs too much time<br>▪ VR-goggles too expensive<br>▪ Don't know how to get VR-goggles<br>▪ It is not pleasant to use<br>▪ Other	Informational question
Did you register on a website for CPR-volunteers?	• Yes, after participation in the study<br>• Yes, but I already was before study participation<br>• No, but I am planning to<br>• No, I don't want to<br>• No, I don't know what that is	Informational question
Did you witness a cardiac arrest following study participation?	• Yes, professionally<br>• Yes, non-professionally<br>• No	Informational question
Did you perform CPR following study participation?	• Yes, professionally<br>• Yes, non-professionally<br>• No	Informational question
**Action taken after study participation - family/acquaintances**		
Did you tell family/acquaintances about participating in the study?	• Don't know<br>• No<br>• Yes<br>▪ 1-10 persons<br>▪ 10-20 persons<br>▪ More than 20	Informational question
Did you tell family/acquaintances about the importance of CPR in general?	• Don't know<br>• No<br>• Yes<br>▪ 1-10 persons<br>▪ 10-20 persons<br>▪ More than 20	Informational question
Did you tell family/acquaintances about the importance of CPR-training?	• Don't know<br>• No<br>• Yes<br>▪ 1-10 persons<br>▪ 10-20 persons<br>▪ More than 20	Informational question
Did your family/acquaintances take action in response to your participation?	• Don't know<br>• No<br>• Yes<br>▪ Look for information on CPR-course<br>▪ Applied for CPR-course<br>▪ Did a CPR-course	Informational question
**Theoretical capability to perform CPR**		
On the Lowlands festival, we did a study to investigate two ways to teach CPR. This is a unique study setting where we gave a CPR-course to over 400 persons. If you read this question entirely, please select "I am not satisfied".	• I am satisfied<br>• I am not satisfied<br>• Neutral	Attention question
Q1: You are on the platform and see a man laying on the floor. What do you do first?	a) Check for safety<br>b) Check for breathing<br>c) Check for response	Knowledge question
Q2: To check if the man is conscious, you have to do the following:	a) Gently shake his shoulders and ask loudly Sir can you hear me?	Knowledge question

© 2022 Nas J et al. *JAMA Network Open.*

	b) Gently shake his head and ask loudly Sir can you hear me?<br>c) Gently shake his shoulders, pinch his arm and scream Sir can you hear me?	
Q3: The man on the platform does not respond. What do you do immediately?	a) Call 112<br>b) Open the airway<br>c) Start chest compressions	Knowledge question
Q4: How would you make sure that the airway of the man is opened?	a) Correct chinlift<br>b) Pinch the nose with 1 hand and place the other hand under the chin to tilt the head<br>c) Put one hand on the forehead to tilt the head backwards	Knowledge question
Q5: If the man is not breathing normally, what do you do first?	a) Start chest compressions<br>b) Put him on his side<br>c) Call 112 and wait for the ambulance to arrive	Knowledge question
Q6: How deep should you perform chest compressions when performing CPR?	a) 5 to 6 cm<br>b) At least 7 cm<br>c) 4 to 5 cm	Knowledge question
Q7: How fast should you perform chest compressions?	a) 2 compressions per second<br>b) 1 compression per second<br>c) 1 compression per 10 seconds	Knowledge question
Q8: When should you stop chest compressions to give mouth-to-mouth ventilations?	a) After 30 compressions<br>b) After 10 compressions<br>c) After about 1 minute of compressions	Knowledge question
Q9: How do you place the AED pads?	a) Figure 1<br>b) Figure 2<br>c) Figure 3	Knowledge question
*This column was not provided to the survey respondent but is included in this supplemental material for reference.		

© 2022 Nas J et al. *JAMA Network Open.*

eTable 2. Baseline Characteristics of Included and Excluded Participants

	Included participants (n=188)	Excluded participants (n=193)	p-value
Female sex (n=379)	115 (61%)	101 (53%)	0.10
Age, years (n=381)	26 (22-32)	25 (22-31)	0.55
Weight, kg (n=378)	71 (64-80)	70 (62-78)	0.16
University education (n=381)	88 (47%)	83 (43%)	0.46
Health care professional (n=380)	50 (27%)	29 (15%)	0.005
Alcohol level, ‰ (n=381)	0.0 (0.0-0.32)	0.0 (0.0-0.37)	0.41
Alcohol level ≥ 0.5 ‰ (n=381)	24 (13%)	39 (20%)	0.05
Drugs or narcotics ≤ 24 hours (n=369)	43 (23%)	50 (27%)	0.42
Previous CPR-course ≤ 2 years (n=358)	34 (19%)	24 (13%)	0.11
Ever witnessed a cardiac arrest (n=381)	28 (15%)	29 (15%)	0.97
CPR-quality parameters			
Compression depth (mm) (n=352)	57 (49-60)	56 (49-59)	0.82
Compression rate (min^{-1}) (n=352)	115 (105-121)	112 (103-120)	0.15

Table showing baseline characteristics of included vs. excluded participants. VR: Virtual reality, CPR: Cardiopulmonary resuscitation.

© 2022 Nas J et al. *JAMA Network Open.*

eTable 3. Comparison of Participants With and Without Informed Consent for Follow-up

	Informed consent for follow-up (n=320)	No informed consent for follow-up (n=61)	p-value
Female sex (n=379)	183 (57%)	33 (55%)	0.73
Age, years (n=381)	26 (22-31)	25 (23-29)	0.31
Weight, kg (n=378)	71 (63-80)	70 (64-77)	0.57
University education (n=381)	144 (45%)	27 (44%)	0.92
Health care professional (n=380)	71 (22%)	8 (13%)	0.11
Alcohol level, ‰ (n=381)	0.00 (0.00-0.33)	0.00 (0.00-0.42)	0.71
Alcohol level ≥ 0.5 ‰ (n=381)	50 (16%)	13 (21%)	0.27
Drugs or narcotics ≤ 24 hours (n=369)	79 (25%)	14 (25%)	0.97
Previous CPR-course ≤ 2 years (n=358)	53 (18%)	5 (9%)	0.08
Ever witnessed a cardiac arrest (n=381)	55 (17%)	2 (3%)	0.005

Table showing baseline characteristics of participants with vs. without informed consent for follow-up. CPR: Cardiopulmonary resuscitation.

© 2022 Nas J et al. *JAMA Network Open.*

eTable 4. Summary of Results With Exclusion of all Health Care Providers

Q	Answer	All participants N=137	Face-to-face N=63	Lifesaver VR N=74	P-value
**Do you feel capable to perform CPR following study participation?**					
	Yes, because of study participation	40 (29%)	23 (37%)	17 (23%)	0.32
	Yes, already before the study	33 (24%)	12 (19%)	21 (28%)	
	Yes, because I did a CPR-training after the study	7 (5%)	3 (5%)	4 (5%)	
	No	57 (42%)	25 (40%)	32 (43%)	
**If you were to witness a cardiac arrest of an unknown person, would you start CPR?**					
	Yes	97 (71%)	47 (75%)	50 (68%)	0.07
	No	6 (4%)	0 (0%)	6 (8%)	
	Don't know	34 (25%)	16 (25%)	18 (24%)	
**If you were to witness a cardiac arrest of an family member or friend, would you start CPR?**					
	Yes	115 (84%)	55 (87%)	60 (81%)	0.17
	No	4 (3%)	0 (0%)	4 (5%)	
	Don't know	18 (13%)	8 (13%)	10 (14%)	
**I would scared to perform CPR**					
	Strongly disagree	4 (3%)	1 (2%)	3 (4%)	0.90
	Disagree	16 (12%)	7 (11%)	9 (12%)	
	Neutral	33 (24%)	15 (24%)	18 (24%)	
	Agree	67 (49%)	31 (49%)	36 (49%)	
	Strongly agree	17 (12%)	9 (14%)	8 (11%)	
**Did you follow an instructor-led CPR-training following study participation?**					
	Yes	17 (12%)	9 (14%)	8 (11%)	0.54
	No	120 (88%)	54 (86%)	66 (89%)	
	I feel capable enough	15 (11%)	4 (6%)	11 (15%)	0.19
	It costs too much time	9 (7%)	4 (6%)	5 (7%)	
	It is too expensive	25 (18%)	14 (22%)	11 (15%)	
	Not yet, but planning to	41 (30%)	22 (35%)	19 (26%)	
	Other	30 (22%)	10 (16%)	20 (27%)	
**Did you tell family/acquaintances about the importance of CPR-training?**					
	Don't know	14 (10%)	5 (8%)	9 (12%)	0.41
	No	42 (31%)	17 (27%)	25 (34%)	
	Yes	81 (59%)	41 (65%)	40 (54%)	
	1-10 persons	68 (50%)	35 (56%)	33 (45%)	0.56
	10-20 persons	9 (7%)	5 (8%)	4 (5%)	
	More than 20	4 (3%)	1 (2%)	3 (4%)	
**Did your family/acquaintances take action in response to your participation?**					
	Don't know	60 (44%)	26 (41%)	34 (46%)	0.85
	No	69 (50%)	33 (52%)	36 (49%)	
	Yes	8 (6%)	4 (6%)	4 (5%)	
	Look for information on CPR-course	6 (4%)	3 (5%)	3 (4%)	1.00
	Applied for CPR-course	2 (2%)	1 (2%)	1 (1%)	1.00
	Did a CPR-course	3 (2%)	0 (0%)	3 (4%)	0.25
**Total score CPR-knowledge test (each correctly answered questions is 1 point)**		7 (6-8)	7 (6-8)	7 (5-8)	0.84

Table showing the summary of results with exclusion of all health care providers for the overall population, face-to-face trained participants and Lifesaver VR-trained participants. P-values are for comparisons between face-to-face and Lifesaver VR-trained participants. CPR: Cardiopulmonary resuscitation, VR: Virtual reality.

© 2022 Nas J et al. *JAMA Network Open.*

eTable 5. Summary of Results With Exclusion of Participants With Alcohol Levels ≥ 0.5 ‰

Q	Answer	All participants N=164	Face-to-face N=89	Lifesaver VR N=75	P-value
**Do you feel capable to perform CPR following study participation?**					
	Yes, because of study participation	44 (27%)	25 (28%)	19 (25%)	0,95
	Yes, already before the study	62 (38%)	34 (38%)	28 (37%)	
	Yes, because I did a CPR-training after the study	7 (4.3%)	4 (5%)	3 (4%)	
	No	51 (31%)	26 (29%)	25 (33%)	
**If you were to witness a cardiac arrest of an unknown person, would you start CPR?**					
	Yes	128 (78%)	73 (82%)	55 (73%)	0,02
	No	6 (4%)	0 (0%)	6 (8%)	
	Don't know	30 (18%)	16 (18%)	14 (19%)	
**If you were to witness a cardiac arrest of an family member or friend, would you start CPR?**					
	Yes	143 (87%)	80 (90%)	63 (84%)	0,09
	No	4 (2%)	0 (0%)	4 (5%)	
	Don't know	17 (10%)	9 (10%)	8 (11%)	
**I would scared to perform CPR**					
	Strongly disagree	9 (6%)	6 (7%)	3 (4%)	0,92
	Disagree	25 (15%)	13 (15%)	12 (16%)	
	Neutral	41 (25%)	23 (26%)	18 (24%)	
	Agree	77 (47%)	40 (45%)	37 (49%)	
	Strongly agree	12 (7%)	7 (8%)	5 (7%)	
**Did you follow an instructor-led CPR-training following study participation?**					
	Yes	24 (15%)	14 (16%)	10 (13%)	0,67
	No	140 (85%)	75 (84%)	65 (87%)	
	I feel capable enough	22 (13%)	8 (11%)	14 (22%)	0,46
	It costs too much time	8 (5%)	5 (7%)	3 (5%)	
	It is too expensive	24 (15%)	13 (17%)	11 (17%)	
	Not yet, but planning to	45 (27%)	27 (36%)	18 (28%)	
	Other	41 (25%)	22 (29%)	19 (29%)	
**Did you tell family/acquaintances about the importance of CPR-training?**					
	Don't know	15 (9%)	7 (8%)	8 (11%)	0,69
	No	54 (33%)	28 (32%)	26 (35%)	
	Yes	95 (58%)	54 (61%)	41 (55%)	
	1-10 persons	79 (48%)	44 (82%)	35 (85%)	0,38
	10-20 persons	10 (6%)	5 (9%)	5 (12%)	
	More than 20	6 (4%)	5 (9%)	1 (2%)	
**Did your family/acquaintances take action in response to your participation?**					
	Don't know	65 (40%)	32 (36%)	33 (44%)	0,57
	No	90 (55%)	52 (58%)	38 (51%)	
	Yes	9 (6%)	5 (6%)	4 (5%)	
	Look for information on CPR-course	6 (4%)	3 (3%)	3 (4%)	1.00
	Applied for CPR-course	3 (2%)	2 (2%)	1 (1%)	1.00
	Did a CPR-course	3 (2%)	0 (0%)	3 (4%)	0.09
**Total score CPR-knowledge test (each correctly answered questions is 1 point)**		7 (6-8)	7 (6-8)	7 (6-8)	0.52

Table showing the summary of results with exclusion of participants with alcohol levels ≥ 0.5 ‰ for the overall population, face-to-face trained participants and Lifesaver VR-trained participants. P-values are for comparisons between face-to-face and Lifesaver VR-trained participants. CPR: Cardiopulmonary resuscitation, VR: Virtual reality.

© 2022 Nas J et al. *JAMA Network Open.*
