# Supplementary material for: Long-term Effect of Face-to-Face vs Virtual Reality Cardiopulmonary Resuscitation (CPR) Training on Willingness to Perform CPR, Retention of Knowledge, and Dissemination of CPR Awareness: A Secondary Analysis of a Randomized Clinical Trial
Source: JAMA Netw Open. 2022 May 19;5(5):e2212964. doi: 10.1001/jamanetworkopen.2022.12964 (PMC9121185; doi:10.1001/jamanetworkopen.2022.12964)
Supplement: Supplement 3. — Data Sharing Statement [file jamanetwopen-e2212964-s003.pdf]

Data Sharing Statement

Nas. Long-term Effect of Face-to-Face vs Virtual Reality Cardiopulmonary Resuscitation (CPR) Training on Willingness to Perform CPR, Retention of Knowledge, and Dissemination of CPR Awareness. *JAMA Netw Open*. Published May 19, 2022.
doi:10.1001/jamanetworkopen.2022.12964

Data
Data available: Yes
Data types: Deidentified participant data
How to access data: j.nas@radboudumc.nl
When available: With publication

Supporting Documents
Document types: None

Additional Information
Who can access the data: Researchers whose proposed use of the data has been approved.
Types of analyses: Analyses that have been approved by the corresponding author.
Mechanisms of data availability: After approval of a proposal.
